# DomainRBF: a Bayesian regression approach to the prioritization of candidate domains for complex diseases

**DOI:** 10.1186/1752-0509-5-55

**Published:** 2011-04-19

**Authors:** Wangshu Zhang, Yong Chen, Fengzhu Sun, Rui Jiang

**Affiliations:** 1 MOE Key Laboratory of Bioinformatics and Bioinformatics Division, TNLIST/Department of Automation, Tsinghua University, Beijing 100084, China; 2 Molecular and Computational Biology Program, University of Southern California, Los Angeles, CA90089, USA

## Abstract

**Background:**

Domains are basic units of proteins, and thus exploring associations between protein domains and human inherited diseases will greatly improve our understanding of the pathogenesis of human complex diseases and further benefit the medical prevention, diagnosis and treatment of these diseases. Within a given domain-domain interaction network, we make the assumption that similarities of disease phenotypes can be explained using proximities of domains associated with such diseases. Based on this assumption, we propose a Bayesian regression approach named "*domainRBF*" (domain Rank with Bayes Factor) to prioritize candidate domains for human complex diseases.

**Results:**

Using a compiled dataset containing 1,614 associations between 671 domains and 1,145 disease phenotypes, we demonstrate the effectiveness of the proposed approach through three large-scale leave-one-out cross-validation experiments (random control, simulated linkage interval, and genome-wide scan), and we do so in terms of three criteria (precision, mean rank ratio, and AUC score). We further show that the proposed approach is robust to the parameters involved and the underlying domain-domain interaction network through a series of permutation tests. Once having assessed the validity of this approach, we show the possibility of *ab initio *inference of domain-disease associations and gene-disease associations, and we illustrate the strong agreement between our inferences and the evidences from genome-wide association studies for four common diseases (type 1 diabetes, type 2 diabetes, Crohn's disease, and breast cancer). Finally, we provide a pre-calculated genome-wide landscape of associations between 5,490 protein domains and 5,080 human diseases and offer free access to this resource.

**Conclusions:**

The proposed approach effectively ranks susceptible domains among the top of the candidates, and it is robust to the parameters involved. The *ab initio *inference of domain-disease associations shows strong agreement with the evidence provided by genome-wide association studies. The predicted landscape provides a comprehensive understanding of associations between domains and human diseases.

## Background

Over the past few decades, remarkable success has been achieved for such traditional gene-mapping approaches as family-based linkage analysis [1,2] and population-based association studies [3,4] in pinpointing genes that are responsible for human inherited diseases [5,6]. Nevertheless, these traditional methods are either only capable of linking diseases with genetic regions that typically contain dozens to hundreds of genes, or usually require carefully selected candidate genes that are biologically related to the disease under investigation [5,6]. Consequently, the development of computational methods for the inference of genes and their protein products that are truly responsible for the disease of interest has been one of the major tasks in human genetics and functional genomics [7-18]. Particularly, a protein typically consists of several structural domains, each of which is closely related to a specific function of the protein. Therefore, the inference of causative genes could be aided by first dividing products of candidate genes into discrete domains with known functions and structural features and then infer the association of these domains to the disease of interest [19-21]. Following this direction, a new protein domain called PAAD has been discovered to be associated with apoptosis, cancer, and autoimmune diseases [19], and a novel domain called G8 has been reported to be linked to polycystic kidney disease and non-syndromic hearing loss [20]. However, most of these discoveries have thus far been made with the assistance of protein sequence analysis and other experimental techniques. Even though such findings are significant, the associations reported are still very sporadic. Therefore, it would be helpful to develop computational methods to directly infer possible associations between domains and human diseases.

It has been shown that deleterious nonsynonymous single nucleotide polymorphisms (nsSNPs) that are responsible for a specific disease of interest may change structures of some protein domains, affect functions of corresponding proteins, and further result in the disease under investigation. Therefore, existing associations between domains and diseases can be constructed by bridging protein domains that contain known deleterious nsSNPs and human diseases with which the nsSNPs are associated [22]. Furthermore, recent advances in computational functional genomics have enabled the large-scale prediction of domain-domain interactions and have led to repositories of known and predicted domain-domain interactions such as DOMINE [23] and InterDom [24,25]. Accordingly, large-scale inference of unknown associations between domains and human diseases can be performed by using these data sources. For example, one of our previous studies [22] adopted the "guilt-by-association" principle [26] to compute scores that quantify the strength of associations between a query disease and candidate domains from domain-domain interaction data and known associations between the query disease and other domains, and then rank candidate domains according to their scores. However, the scope of application of this approach is limited because the "guilt-by-association" principle relies on known associations between the query disease and domains to infer novel associations for the query disease. Under these conditions, the method cannot be applied to diseases whose genetic bases are completely unknown.

Recent studies on the modular nature of human genetic diseases have shown that diseases share common clinical characteristics are often caused by functionally related genes [16,27]. With the application of text mining techniques, it has also been possible to calculate pair-wise similarities for most human disease phenotypes [28]. With these advances, various methods have been proposed to prioritize candidate genes through the combined use of disease phenotype similarity and gene proximity [7,8,29-32]. Inspired by the successes of these methods, we propose in this paper to infer associations between domains and human disease phenotypes based on the assumption that phenotypically similar diseases are caused by functionally related domains. More specifically, we resort to a linear regression framework to model the relationship between a domain proximity profile and a phenotype similarity profile, and we develop a Bayesian regression approach, called *domainRBF *(domain Ranking with Bayes Factor), to calculate Bayes factors that quantify the strength of associations between corresponding domain proximity profiles and phenotype similarity profiles.

We compile a set of known domain-disease associations using the Pfam database [33] and annotations of nsSNPs in the UniProt database [34,35], extract a domain-domain interaction network from the DOMINE database [23] as well as the InterDom database [24,25], and then download a pre-calculated phenotype similarity network [28]. Using these data, we show that domain proximities calculated from a domain-domain interaction network do, indeed, imply phenotype similarities of diseases. We next validate the approach and evaluate its performance using three criteria: precision, mean rank ratio, and AUC score. To accomplish this, we apply three large-scale leave-one-out cross-validation experiments against random control, simulated linkage interval, and genome-wide scan with two domain proximity measures: diffusion kernel and shortest path with Gaussian kernel. Results show that the proposed approach can successfully recover known associations between domains and human diseases. We further show the robustness of this approach to the parameters involved and the underlying domain-domain interaction network through a series of permutation tests. Having successfully assessed the validity and robustness of this approach, we can then infer domain-disease in an *ab initio *way and illustrate the strong agreement of the inference results with evidence of genome-wide association studies for four common human diseases, including type 1 diabetes, type 2 diabetes, Crohn's disease, and breast cancer. We further demonstrate the possibility of inferring gene-disease associations from domain-disease associations. Finally, we calculate a genome-wide landscape of associations between 5,490 domains and 5,080 human diseases using all known domain-disease associations, and we provide a freely accessible website for this resource.

## Methods

### Overview of the DomainRBF approach

We ground the inference of domains that are associated with human inherited diseases on a set of known domain-disease associations that are compiled from the Pfam database [33] and annotations of nsSNPs in the UniProt database [34,35], a domain-domain interaction network extracted from the DOMINE database [23] and the InterDom database [24,25], as well as a pre-calculated phenotype similarity network containing pair-wise similarity scores among more than 5,000 human genetic disease phenotypes in the OMIM database [28].

**Figure 1:**
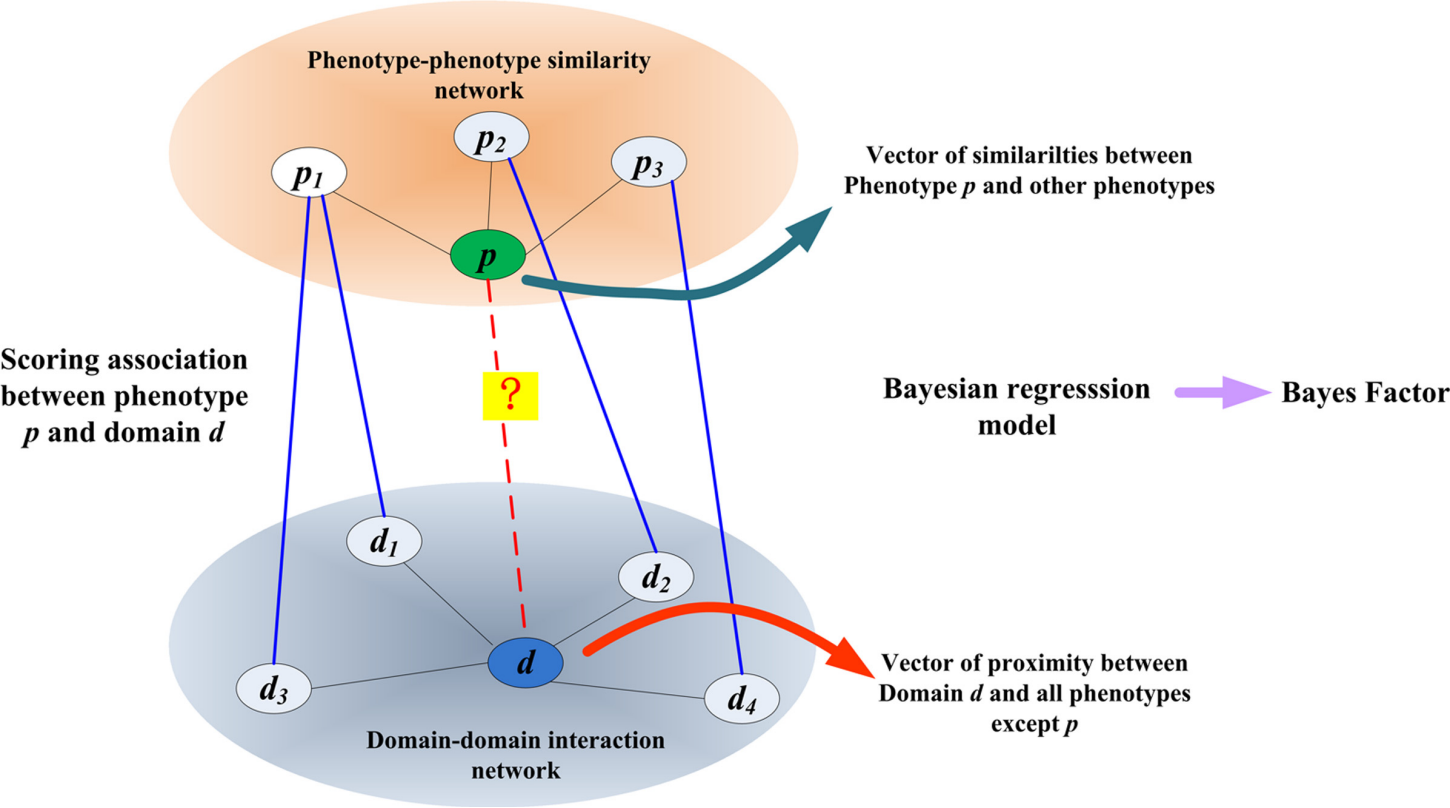
**Scheme of the proposed domainRBF approach**. Texts in addition to pink arrows denote the pipeline of the domainRBF approach.

Based on the assumption that phenotypically similar diseases are caused by functionally related domains, we propose a linear regression framework to model the relationship between a domain proximity profile and a phenotype similarity profile, and we resort to a Bayesian approach to solve the linear regression model. As shown in Figure 1 (inspired by Ideker and Sharan [36]), given a query phenotype *p *and the pre-calculated pair-wise similarity scores between phenotypes, we extract scores between the query phenotype and all other phenotypes that have at least one associated domain and obtain a phenotype similarity profile for the query phenotype. On the other hand, for a query domain *d *in a set of candidate domains, we resort to the domain-domain interaction network to calculate proximity scores of the query domain to all domains that are known to be associated with some phenotypes and further calculate a domain proximity profile. With these two profiles, we propose a Bayesian regression approach called *domainRBF *(domain Ranking with Bayes Factor) to calculate a Bayes factor that quantifies the strength of association between the query domain and the query phenotype, using the phenotype similarity profile as the response variable and the domain proximity profile as the predictor variable. Finally, we rank candidate domains according to their corresponding Bayes factors and obtain a rank list of the candidates.

### Data sources

#### Domain-disease associations

A domain is defined as associated with a disease if the domain contains at least one nonsynonymous single nucleotide polymorphism (nsSNP) associated with the disease [22]. Therefore, associations between domains and diseases are obtained by combining known associations between nsSNPs and diseases as well as relationships between protein and domains.

Known associations between nsSNPs and diseases are obtained from annotations of nsSNPs in the UniProt database [34,35], in which nsSNPs are classified into three categories: disease, polymorphism, and unclassified. In version 57.15 (released on March 2, 2010) of this database, 23,372 nsSNPs belong to the disease category, 36,303 belong to the polymorphism category, and the remaining 2,019 nsSNPs are currently unclassified. For each of the nsSNPs in the disease category, the entry ID of the specific disease in the OMIM database is also provided. Consequently, we obtain 19,552 associations between 19,552 nsSNPs and 1,592 diseases.

Relationships between human proteins and domains are obtained from the Pfam database [33], which provides a large collection of both high quality protein domain families (Pfam-A) and low quality protein domain families (Pfam-B). In version 24.0 of the Pfam-A collection (released in October 2009), 11,912 domain families that cover more than 75.15% of known proteins are collected. Using this data source, we obtain 96,276 relationships between 4,324 domains and 66,498 human proteins.

Using the above data sources and having defined a domain as associated with a disease if the domain contains at least one nsSNP associated with the disease, we are able to establish 1,614 associations between 671 domains and 1,145 diseases.

#### Domain-domain interaction networks

Our inference of domain-disease associations is based on domain-domain interaction networks extracted from the DOMINE [23] and InterDom [24,25], two of the most widely-used databases of known and predicted domain-domain interactions.

The latest version of DOMINE (released in February 2008) contains a total of 20,513 domain-domain interactions, out of which 4,349 (gold-standard positives) are inferred from PDB entries (the union of the sets of interactions from iPfam [37] and 3did [38,39]), and 17,781 are predicted by at least one computational approach of 8 different computational approaches using Pfam domain definitions. Of the 17,781 predicted interactions, there are 3,143 high-confidence predictions (predicted by ME [40] or at least two different approaches), 729 medium-confidence predictions (hetero-domain interactions in which both domains have the same annotations in the biological process of the gene ontology), and 13,909 remaining low-confidence predictions [23].

The latest version of InterDom (released in July 31, 2007) contains a total of 148,938 domain-domain interactions, out of which 7,718 are inferred from PDB entries [41], 143,820 are inferred from BIND [42] and DIP [43] entries, and 4,631 are inferred from the domain fusion hypothesis. InterDom further uses a probabilistic scoring system to give confidence scores to domain interactions that are derived independently by multiple methods from different data sources. Finally, interactions with 90%, 75%, 50%, and 25% confidence levels are provided [24,25].

In our work, we use two domain-domain interaction networks extracted from these data. First, we discard singletons in the PDB part of the DOMINE database [23] and obtain a small network that is composed of 2,285 interactions between 1,971 domains (2.32 interactions per domain on average). Second, we combine 37,177 interactions whose confidence scores are at least 90% in the InterDom database and all interactions in the DOMINE databases to obtain a large domain-domain interaction network that is composed of 48,778 interactions between 5,490 domains (17.77 interactions per domain on average).

#### The phenotype similarity network

The phenotype similarity network of human diseases is a fully connected network obtained from an earlier work of van Driel *et al*. [28], in which the pair-wise relationships between 5,080 human genetic diseases from the OMIM database are mapped. Briefly, van Driel *et al*. use the anatomy (A) and the disease (C) sections of the medical subject headings vocabulary (MeSH) to extract terms from the OMIM database, thus providing a standard way of presenting the OMIM records as corresponding phenotype feature vectors. As a result, each disease phenotype is characterized by a vector of standardized and weighted phenotypic feature terms mapped from corresponding OMIM records in the full text (TX) and clinical synopsis (CS) fields. Then, for each pair of disease phenotypes, a similarity score is calculated by the cosine of their feature vector angle. The reliability of the phenotype similarity score has been tested [28], showing that these similarities are positively correlated with a number of measures of gene functions. The final phenotype similarity network contains pair-wise similarity scores for 5,080 OMIM records, covering a majority of recorded human disease phenotypes.

### The DomainRBF model

Given the phenotype similarity network, we use *y*_*pp' *_to denote the similarity score between a query disease phenotype *p *and another disease phenotype *p'*. We further define the phenotype similarity profile for disease phenotype *p *as , i.e., the similarities between the disease phenotype *p *and all *m *disease phenotypes *p*_*1*_, *p*_*2*_, ..., *p*_*m *_that have at least one associated domain.

On the other hand, given a domain-domain interaction network of *n *nodes, we calculate the proximity between two domains using two measures: (1) shortest path with Gaussian kernel (SG) and (2) diffusion kernel (DK). The shortest path proximity between two domains *u *and *v*, *SP*(*u,v*), is defined as the length of the shortest path between the two domains. Using the Gaussian kernel, the proximity distance measure *SG *(*u*, *v*) is obtained as *SG*(*u,v*) *= *exp{*-β*(*SP*(*u,v*))^2^}, where *β *is a free parameter. The diffusion kernel for the network is defined as **K **= (*k*_*uv*_)_*n*__×__*n *_= *e*^-^^*γL*^, where 0 < γ < 1 is a free parameter that controls the magnitude of diffusion. The matrix **L **= **D **- **A **is the Laplacian of the network, where **D **is a diagonal matrix containing node degrees, and **A **is the adjacency matrix of the domain-domain interaction network. With the diffusion kernel **K **= (*k*_*uv*_)_*n*__×__*n*_, we define the diffusion proximity of two domains *u *and *v *as *DK*(*u*,*v*) = *k*_*uv*_, i.e., the corresponding element in the diffusion kernel. Then, let *x*_*dd' *_denote the proximity between domains *d *and *d' *in the domain-domain interaction network, and let *D*(*p*) denote the set of domains known to be associated with a phenotype *p*. We define the proximity between domain *d *to disease phenotype *p *as the summation of proximity scores between domain *d *and all domains known to be associated with disease phenotype *p*, i.e., *x*_*dp *_= ∑_*d*__'∈__*D*__(__*p*__) _*x*_*dd*__'_. We further define the domain proximity profile for domain *d *as .

Then, given a query disease phenotype *p *and a query domain *d*, we explain the phenotype similarity profile **y**_*p *_using domain proximity profile **x**_*d *_via a linear regression model

where **y **= **y**_*p *_is the response vector, **X **= (**1**,**x**_*d*_) the design matrix, **β ***= *(*β*_0_, *β*_1_)^*T *^the coefficient vector, and **ε **= (*ε*_1_,..., *ε*_*m*_)^*T *^the residual vector. Note that the first column of the design matrix being 1s for the purpose of incorporating the intercept. We propose to solve this linear regression model using a Bayesian approach. We choose to take a Bayesian approach because it provides a natural way to consider the uncertainty in estimated parameters, and it provides Bayes factor, a measure of the strength of evidence for an association, which is defined as the ratio of marginal likelihoods for **y **conditional on **X **under the alternative and the null hypothesis, respectively, as described below.

For the alternative model, we assume that **y **conditional on **X **is subject to a normal distribution, as

with residuals independent and identically distributed, following normal density with mean 0 and variance σ^2^. We set conjugate prior distributions for **β **and σ^2^, as

and

where **μ**_0 _*= *(*μ*_0_, *μ*_1_)^*T *^is composed of prior means, and σ^2^**Σ**_0 _prior variances with **Σ**_0 _= diag(σ_*μ*_^2^,σ_1_^2^) being a diagonal matrix. The joint distribution of all random quantities **y**, **β**, and σ^2 ^is then given as

Integrating out **β **and σ^2^, we obtain the marginal likelihood of **y **given **X **as

where *n*_*n *_= *n *+ *n*_0 _and *n*_*n *_σ_n_^2 ^= *n*_0_σ_0_^2 ^+ **y**^*T*^**y **+ **μ**_0_^*T *^**Σ**_0_^-1^**μ**_0_-**μ**_*n*_^*T *^**Σ**_*n*_^-1^**μ**_*n *_with **Σ**_n _= (**X**^*T *^**X **+ **Σ**_0_^-1^)^-1 ^and **μ**_*n *_= **Σ**_n _(**X**^*T *^**y + Σ**_0_^-1^**μ**_0_).

On the other hand, for the null model, where **y **is independent of **X**, the marginal likelihood of **y **can be derived in a similar way, as

where , and .

Then, the Bayes factor BF is the ratio of *p*_1_(**y**|**X**) and *p*_0_(y), as

Following the literature [44], we take the limit +∞ for σ_*μ*_^2 ^and 0 for both *n*_0 _and σ_0_^2^, and we obtain the limit value of the Bayes factor as

For simplicity, we further set **μ**_0 _= **0 **as in the literature [44], and we set σ_1_^2 ^= 1 as the default setting in this paper, although the effect of these parameters are also studied.

Note that before the construction of the Bayesian regression relationship between **y**_*p *_and **x**_*d*_, we apply an inverse-normal transform to **y**_*p *_to guarantee that the responsive variable is normally distributed. As illustrated in [45,46], the transform formula we use is:

where *r*_*i *_is the rank of  in the vector **y**_*p*_, *m *the length of **y**_*p*_, and Φ the cumulative distribution function of the standard normal distribution.

### Validation methods and evaluation criteria

On the basis of the domain-domain interaction network and known associations between protein domains and disease phenotypes, we proceed to validate how well the proposed approach performs in recovering these known associations. We adopt three large scale leave-one-out cross-validation experiments for this purpose.

First, in the validation of random controls, we prioritize domains that are known to be associated with disease phenotypes (i.e., disease domains) against randomly selected control domains. Specifically, in each run of the validation, we select an association between a domain and a disease phenotype, assume that the association is unknown, and prioritize the domain against a set of 99 randomly selected control domains.

Second, in the validation of simulated linkage intervals, we prioritize domains that are known to be associated with disease phenotypes (i.e., seed domains) against domains that are located around the seed domains. Specifically, in each run of the validation, we select an association between a domain and a disease phenotype, assume that the association is unknown, and prioritize the domain against a set of control domains that are located within 10 Mbp upstream and downstream of this domain.

Third, in the validation of genome-wide scan, we prioritize seed domains against all known domains. Specifically, in each run of the validation, we select an association between a domain and a disease phenotype, assume that the association is unknown, and prioritize the domain against all other domains in the domain-domain interaction network.

In each of the above leave-one-out cross-validation experiments, we repeat the validation run for every known association between a domain and a disease phenotype, and we are able to obtain a number of ranking lists. We further normalize the ranks by dividing them by the total number of candidate domains in the rankling list to obtain rank ratios and calculate the values of three criteria to measure the performance of a prioritization method.

The first criterion is termed precision. We consider a prediction as successful if the known disease domain is ranked at the top (with rank 1). Then, the proportion of successful predictions among all predictions is defined as the precision. Obviously, a high precision suggests that a method has high prediction power. The second criterion is termed mean rank ratio, which is simply the average of rank ratios for all known disease domains in a cross-validation experiment. This criterion provides a summary of the ranks of all domains that are known to be associated with disease phenotypes, and the smaller the mean rank ratio, the better a method. The third criterion is termed AUC, which is the area under the receiver operating characteristic curve (ROC). Given a list of rank ratios and a predefined threshold, we define the sensitivity as the percentage of disease domains that are ranked above the threshold and the specificity as the percentage of control domains that are ranked below the threshold. By varying the threshold values, we are able to plot a receiver operating characteristic curve, which shows the relationship between sensitivity and 1-specificity. Calculating the area under the ROC curve (AUC), we are able to obtain the AUC score, which provides an overall measure for the performance of the prioritization approach.

## Results

### Domain proximity implying phenotype similarity

The DomainRBF approach is based on the assumption that similarities of disease phenotypes can be explained by proximities of domains associated with the phenotypes within a domain-domain interaction network via a regression model. In order to validate this assumption, we discard singletons in the PDB part of the DOMINE database [23] and obtain a domain-domain interaction network that is composed of 2,285 interactions between 1,971 domains. Focusing on these domains, we obtain 1,066 associations between 763 phenotypes and 378 domains. Then, we calculate a Bayes factor for each of these associations, and run a Wilcoxon signed rank test to check whether the resulting Bayes factors are significantly greater than 1 (the random case). Results show that the *p*-value is smaller than 2.2 × 10^-16^, indicating that the similarities of disease phenotypes have a strong relationship with the proximities of associated domains.

**Figure 2:**
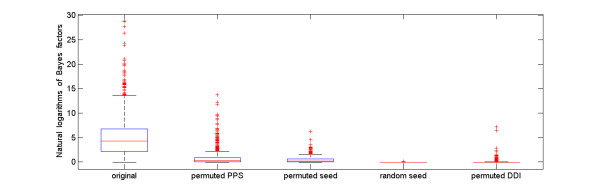
**Bayes factors of the original and permuted data**. "original", "permuted PPS", "permuted seed", "random seed", and "permuted DDI" denote the results obtained using the original data, permuted phenotype similarity profile, permuted domain-disease associations, randomly selected seed domains, and permuted domain-domain interaction network, respectively. The small domain-domain interaction network composed of the PDB part of the DOMINE database and the diffusion kernel are used to obtain the results.

To further substantiate this point, we perform a series of permutations towards disease-disease, domain-disease, and domain-domain relationships. First, we break the disease-disease relationship by permuting the phenotype similarity profile. Second, we break the domain-disease relationship by two methods: (1) permuting domain-disease associations and (2) replacing domains in known disease-domain associations with randomly selected domains. Third, we break the domain-domain relationship by permuting connections in the underlying domain-domain interaction network, while keeping node degrees and recalculating the diffusion kernel. For each of the above permutations, we calculate Bayes factors of disease domains and present the results in Figure 2, which shows that the median of Bayes factors based on the original data is much higher than the medians obtained from the different permuted relationships, as described above.

We also perform similar studies using the large domain-domain interaction network (48,778 interactions between 5,490 domains) that includes the entire DOMINE database [21] and the high-confidence part of the InterDom [22,23] database. Results show that Bayes factors for known domain-disease associations are also significantly greater than 1, while the *p*-value of the Wilcoxon signed rank test is smaller than 2.2 × 10^-16^. We further perform a series of permutation tests and present the results [Additional file 1: Supplemental Figure S1]. Based on these comprehensive studies, our hypothesis has been clearly demonstrated: that similarities between diseases can be explained by the proximities of domains associated with such diseases within a given domain-domain interaction network. In other words, domain proximity implies phenotype similarity.

### Performance of the DomainRBF approach

Since interactions from the PDB entries have the highest confidence of domain-domain interactions, we first test the validity of our approach on the PDB part of the DOMINEdatabase [23]. We implement three large-scale leave-one-out cross-validation experiments against random controls, simulated linkage intervals and genome-wide scan, respectively, each on the basis of two distance measures: diffusion kernel (DK) and shortest path with Gaussian kernel (SG).

**Figure 3:**
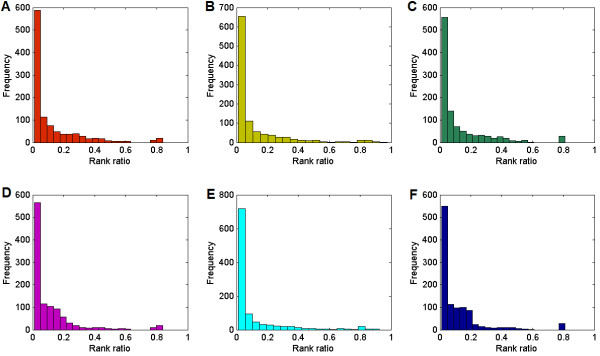
**Histograms of rank ratios for domains known to be associated with diseases**. (A) Results for shortest path with Gaussian kernel, against random controls. (B) Results for shortest path with Gaussian kernel, against linkage intervals. (C) Results for shortest path with Gaussian kernel, against genome-wide scan. (D) Results for diffusion kernel, against random controls. (E) Results for diffusion kernel, against linkage intervals. (F) Results for diffusion kernels, against genome-wide scan. The small domain-domain interaction network composed of the PDB part of the DOMINE database and the diffusion kernel are used to obtain the results.

For each of the three validation experiments, using either the diffusion kernel or the shortest path with Gaussian kernel, we draw a histogram of rank ratios for the entire 1,066 known associations, as shown in Figure 3. From the figure we see that rank ratios are concentrated mostly within the interval of the first few bins, and as the rank ratios increase, corresponding frequencies all take a general trend of declination. In other words, the proposed approach is capable of ranking domains known as associated with some disease phenotypes among the top of the candidates.

We then assess the performance of the proposed approach using the three criteria (mean rank ratio, precision, and AUC score) and summarize the results in Table 1. First, we can see from these results that the domainRBF approach can successfully recover the associations between protein domains and human disease phenotypes. For example, in the cross-validation for random controls, the precisions are greater than 26%, the mean rank ratios are less than 12%, and the AUC scores are greater than 88%. In the cross-validation for linkage intervals, the precisions are greater than 23%, the mean rank ratios are less than 12%, and the AUC scores are greater than 89%. In the cross-validation for genome-wide scan, the precisions are greater than 5%, the mean rank ratios are less than 12%, and the AUC scores are greater than 88%. We therefore conclude that the domainRBF approach is effective in the identification of domains that are associated with human disease phenotypes.

**Table 1:** Results of leave-one-out cross-validation experiments on the small network.

		Random Control (%)		Linkage Interval (%)		Genome-wide Scan(%)	
		
		***R***^**2**^	BF	***R***^**2**^	BF	***R***^**2**^	BF
Precision	SG	26.11 (0.66)	26.56 (0.85)	21.69	23.79	6.29	5.25
	DK	26.25 (0.63)	28.67 (0.72)	19.60	31.20	7.13	5.53
Mean Rank Ratio	SG	17.31 (0.11)	11.99 (0.05)	18.24	11.19	16.49	11.17
	DK	17.82 (0.13)	10.75 (0.09)	19.09	9.73	17.03	9.91
AUC	SG	83.51 (0.10)	88.80 (0.05)	82.81	89.94	83.60	88.85
	DK	83.01 (0.12)	90.04 (0.11)	81.95	90.18	83.06	90.11

The small network indicates the domain-domain interaction network that is composed of the PDB part of the DOMINE database. *R*^2 ^denotes the ordinary non-Bayesian linear regression approach (using R-square as scores for candidate domains). BF denotes the domainRBF approach (using Bayes factors as scores for candidate domains). SG denotes shortest path with Gaussian kernel. DK denotes diffusion kernel. Results for random controls are mean (standard deviation) of 10 validation runs.

Second, we conjecture from these results that the diffusion kernel measure is slightly better than the shortest path measure with Gaussian kernel, because the mean rank ratios obtained using the diffusion kernel are in general smaller, and the precisions and AUC scores are in general larger, than those obtained using the shortest path with Gaussian kernel. This phenomenon might be explained by the fact that diffusion kernel is a global network-distance measure. As such, the distance between two domains not only depends on the relative location of the candidate domain to all other domains (as the shortest path with Gaussian kernel does), but also relies on the graph structure of the entire network. Thus, for interaction networks with different graph structure, two nodes with the same shortest path distance usually have different diffusion kernel distance, and it is possible that this difference makes the diffusion kernel distance more reasonable and precise in the description of similarities between two domains in the interaction network. This point has also been explicitly illustrated in literature [29].

**Figure 4:**
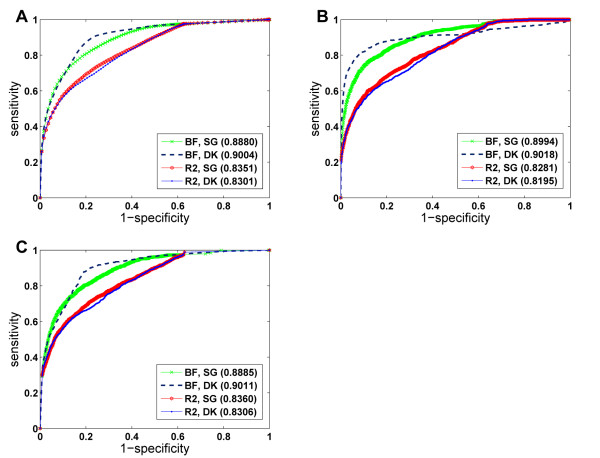
**ROC curves of the leave-one-out cross-validation experiments**. (A) Results for random controls. (B) Results for linkage intervals. (C) Results for genome-wide scan. BF: the domainRBF approach (using Bayes factors as scores for candidate domains). *R*^2^: the ordinary non-Bayesian linear regression approach (using R-square as scores for candidate domains). SG: shortest path with Gaussian kernel. DK: diffusion kernel. Numbers in the parentheses are AUC scores of the corresponding ROC curves. The small domain-domain interaction network composed of the PDB part of the DOMINE database is used to obtain the results.

Third, we conjecture from these results that the domainRBF approach with some proper defined priors can achieve higher performance than the non-Bayesian linear regression method. We compare the performance of the (Bayesian) domainRBF approach with the (non-Bayesian) ordinary linear regression method through the three large-scale leave-one-out cross-validation experiments, and we also list the results in Table 1. Although both approaches can successfully recover the associations between protein domains and human disease phenotypes, the results show that the domainRBF approach can achieve better performance than the ordinary linear regression approach in most cases. For example, in all three cross-validation experiments, the domainRBF approach can achieve higher precisions (with only two exceptions for genome-wide scan), smaller mean rank ratios (for at least 5.32%), and larger AUC scores (for at least 3.19%). When looking at the ROC curves (Figure 4), we see that the curve of the domainRBF approach climbs much faster towards the upper left corner of the plot than does that of the ordinary linear regression approach, suggesting that the Bayesian domainRBF approach is superior to the non-Bayesian ordinary linear regression method.

### Robustness of the DomainRBF approach

#### Effects of network interactions

**Figure 5:**
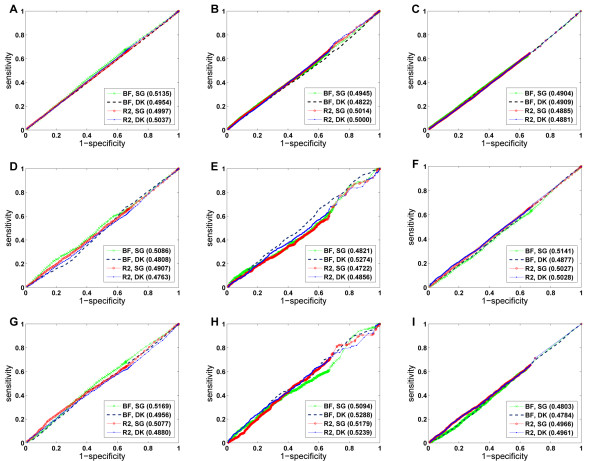
**ROC curves of the leave-one-out cross-validation experiments on shuffled data**. (A) Results for random controls with domain-domain interactions shuffled. (B) Results for linkage intervals with domain-domain interactions shuffled. (C) Results for genome-wide scan with domain-domain interactions shuffled. (D) Results for random controls with known domain-phenotype associations shuffled. (E) Results for linkage intervals with known domain-phenotype associations shuffled. (F) Results for genome-wide scan with known domain-phenotype associations shuffled. (G) Results for random controls with phenotype similarity profiles shuffled. (H) Results for linkage intervals with phenotype similarity profiles shuffled. (I) Results for genome-wide scan with phenotype similarity profiles shuffled. The small domain-domain interaction network composed of the PDB part of the DOMINE database and the diffusion kernel are used to obtain the results.

The above validation results suggest that the domainRBF approach can successfully prioritize candidate domains and put the domain that is truly associated with the query disease phenotype at the top of the candidates. However, it is still necessary to determine whether the correct prioritization of disease domains is due to the connectivity information that includes in the domain-domain interactions, domain-phenotype associations, and phenotype-phenotype similarities. To accomplish this, we artificially destroy informative interactions in the above three networks and see what performances will turn out. It is expected that both the mean rank ratios and the AUC scores will be around 50%, together with very low precisions. With this understanding, we perform three permutation experiments: 1) shuffling interactions among domains while fixing the node degree (number of direct neighbours) distribution of the entire interaction network, 2) shuffling interactions among domain-phenotype associations while fixing the number of associated domains for each of the phenotypes, and 3) shuffling the phenotype-phenotype similarity while fixing the distribution of phenotype similarities, respectively. Then we repeat the leave-one-out cross-validation experiments using the shuffled networks, which contain no informative interactions among domains, among domain and phenotypes, or among phenotypes, respectively. As shown in Figure 5, the results obtained are generally consistent with our expectation in that AUC scores are all around 50%. We therefore conclude that the successful prioritization of candidate domains is indeed due to the informative interactions among domains that are included in the domain-domain interaction network.

#### Effects of different domain-domain interaction networks

We notice that the two compiled domain-domain interaction networks have different properties. For example, the average degree of the smaller network that includes only PDB entries is 2.32, while that of the larger network that includes predicted interactions from both DOMINE and InterDom is 17.77. It is possible that many predicted interactions may actually be noise and thus negatively affect the prioritization of disease domains. Hence, it is necessary to validate the robustness of the proposed approaches to the underlying domain-domain interactions. For this purpose, we implement the same validation process based on the large compiled domain-domain interaction network that is composed of all interactions in the DOMINE database and high-confidence interactions in the InterDom database. Results are presented in Table 2, from which we can see that the performances of the domainRBF approach using the large domain-domain interaction network that includes the entire DOMINE database and the high-confidence interactions in the InterDom database are generally somewhat inferior to those using the PDB part of the DOMINE database. For instance, when using the domainRBF approach, the disparity of precisions, mean rank ratios and AUCs are all within the scope of 10 percent. We then conjecture from these results that the proposed domainRBF approach is quite robust to the possible noise in the domain-domain interaction network.

**Table 2:** Results of leave-one-out cross-validation experiments on the large network.

		Random Control (%)		Linkage Interval (%)		Genome-wide Scan (%)	
		
		***R***^**2**^	BF	***R***^**2**^	BF	***R***^**2**^	BF
Precision	SG	14.24 (0.37)	18.64 (0.66)	18.54	21.23	2.17	2.35
	DK	16.80 (0.36)	22.32 (0.60)	17.36	27.56	2.42	3.47
Mean Rank Ratio	SG	27.27 (0.07)	19.59 (0.09)	21.89	18.07	26.53	18.79
	DK	26.07 (0.11)	14.96 (0.07)	20.21	14.12	25.34	14.11
AUC	SG	73.41 (0.07)	81.15 (0.09)	78.57	81.76	73.38	81.09
	DK	74.61 (0.11)	85.82 (0.07)	82.36	86.68	74.55	85.72

The large network indicates the domain-domain interaction network that is composed of the entire DOMINE database and high-confidence interactions in the InterDom database. *R*^2 ^denotes the ordinary non-Bayesian linear regression approach (using R-square as scores for candidate domains). BF denotes the domainRBF approach (using Bayes factors as scores for candidate domains). SG denotes shortest path with Gaussian kernel. DK denotes diffusion kernel. Results for random controls are mean (standard deviation) of 10 validation runs.

#### Effects of parameters in the distance measures

**Figure 6:**
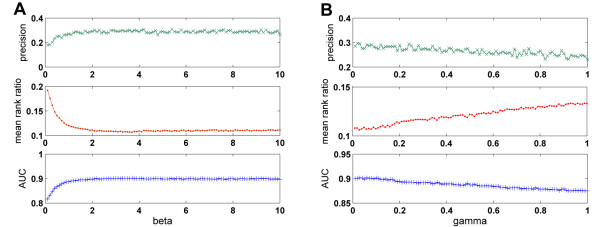
**Effects of parameters on distance measures**. (A) Influence of *β *(from 0.1 to 10 with step 0.1) on shortest path with Gaussian kernel. (B) Influence of *γ *(from 0.01 to 1 with step 0.01) on diffusion kernel. The small domain-domain interaction network composed of the PDB part of the DOMINE database is used to obtain the results.

We further notice that the parameter *β *in the shortest path measure with Gaussian kernel and the parameter *γ *in the diffusion kernel are free parameters that need to be pre-determined (see Materials and Methods for details). In the above cross-validation experiments we set these parameters as 1 and 0.05, respectively, for simplicity. However, it is necessary to show whether the prioritization methods are sensitive to these parameters. For this purpose, we select several values across the range of these parameters, perform the cross-validation experiments, and see how the results change accordingly. We take the prioritization results using the domainRBF approach against random controls (in Table 1) as an example to illustrate the influence of *β*. Since this parameter ranges from 0 to +∞, we perform a grid search of this parameter by changing it from 0.1 to 10 with step 0.1 and see the effect, as reflected in the change of precision, mean rank ratio, and AUC score as shown in Figure 6(A). From the curve we can see that when *β *changes from 0.1 to 1, there is an obvious upward climb for the three criteria, while after the point *β *= 1.0 (precision = 26.56%, mean rank ratio = 11.99%, and AUC score = 88.80%), the values in the curve becomes fairly stable. Even so, we find that the peak performance is obtained at *β *= 3.7 (precision = 28.89%, mean rank ratio = 10.67%, and AUC score = 90.20%), and the worst performance is obtained at *β *= 0.1 (precision = 18.01%, mean rank ratio = 19.23%, and AUC score = 81.63%). From these results, we conclude that the prioritization methods are not sensitive to this free parameter when *β *is greater than 1. Similarly, we find that the prioritization methods are not sensitive to the free parameter *γ *when it is smaller than 0.15 (data not shown). The corresponding changes in precision, mean rank ratio, and AUC score are shown in Figure 6(B). We find that the peak performance is obtained at *γ *= 0.03 (precision = 29.55%, mean rank ratio = 10.61%, and AUC score = 90.16%), and the worst performance is obtained at *γ *= 0.93 (precision = 24.86%, mean rank ratio = 13.34%, and AUC score = 87.46%). From the results, we can see that the proposed approach is quite robust when *β *in the shortest path with Gaussian kernel is greater than 1 or when *γ *in the diffusion kernel is smaller than 0.15.

#### Effects of parameters in the domainRBF approach

Besides the two parameters in the distance measures, there are also four parameters in the domainRBF approach that need to be pre-determined, namely **μ**_0_, σ_1_^2^, *n*_0_, and σ_0_^2^, all of which are included in the priors of the domainRBF approach (see Materials and Methods for details). In the real implementation we set **μ**_0 _= **0**, *n*_0 _= 0, and σ_0_^2 ^= 0, for the reason explained in the literature [44], and we set σ_1_^2 ^= 1, for simplicity. Therefore, we only need to test the robustness of the approach when different values of σ_1_^2 ^are used. To achieve this objective, we set σ_1_^2 ^as 0.001, 0.01, 0.1, 1, 10, and 100, respectively, and we apply the approach to the same cross validation process. We list the results in Table 3, which shows that when σ_1_^2 ^is smaller than 1, the domainRBF approach is quite robust to the change of σ_1_^2^, with the change of precision within 1.12%, change of mean rank ratios within 0.53%, and change of AUC scores within 0.54%. On the other hand, when σ_1_^2 ^is larger than 1, the decrease in performances becomes slightly conspicuous, but remains within the scope of 3.04% for precisions, 2.22% for mean rank ratios and 1.77% for AUC scores. Hence we can see that our domainRBF approach is generally robust to the change of parameters.

**Table 3:** Effects of parameters based on leave-one-out cross-validation experiments.

Criteria	***σ***_**1**_^**2**^	Random Control (%)	Linkage Interval (%)	Genome-wide Scan (%)
Precision	0.001	28.87 (0.70)	31.49	4.41
	0.01	29.02 (1.17)	31.77	4.78
	0.1	29.39 (0.60)	31.36	5.07
	1	28.67 (0.72)	31.20	5.53
	10	27.76 (0.62)	29.73	5.35
	100	28.59 (1.18)	28.16	5.35

Mean Rank Ratio	0.001	10.23 (0.07)	9.83	9.38
	0.01	10.25 (0.11)	9.98	9.41
	0.1	10.41 (0.07)	9.49	9.54
	1	10.75 (0.09)	9.73	9.91
	10	11.14 (0.10)	10.92	10.27
	100	12.08 (0.10)	11.95	11.24

AUC	0.001	90.58 (0.10)	90.14	90.55
	0.01	90.56 (0.14)	90.40	90.61
	0.1	90.40 (0.10)	90.22	90.40
	1	90.04 (0.11)	90.18	90.11
	10	89.69 (0.10)	89.23	89.86
	100	88.74 (0.14)	88.41	88.98

The small domain-domain interaction network composed of the PDB part of the DOMINE database and the diffusion kernel are used to obtain the results. Results for random controls are mean (standard deviation) of 10 validation runs.

#### Effects of seed domain-disease associations

In order to test the influence of the size of seed, or known associations on the prioritization results, we select at random 100%, 90%, 80%, 70%, 60%, and 50% of the original seed associations, respectively, and we repeat the leave-one-out validation processes. We only calculate the performance using the domainRBF approach based on diffusion kernel measure, and we choose the PDB part of the DOMINE database as the domain-domain interaction network. Results show that with the percentage of seed associations decreases from 100% to 50%, performance also slightly decreases in terms of precision, mean rank ratio and AUC score, despite some exceptions (see Table 4). For example, in the cross-validation for random controls, the changes of precisions are no more than 2.99%, the changes of mean rank ratios are no more than 2.72%, and the changes of AUC scores are no more than 2.73%. In the cross-validation for linkage intervals, the changes of precisions are no more than 2.73%, the changes of mean rank ratios are no more than 0.75%, and the changes of AUC scores are no more than 0.78%. In the cross-validation for genome-wide scan, the changes of precisions are no more than 1.95%, the changes of mean rank ratios are no more than 1.04%, and the changes of AUC scores are no more than 0.95%. From these results, we conclude that the prioritization methods are not sensitive to the size of seed associations in our problem.

**Table 4:** Effects of seed domain-disease associations based on leave-one-out cross-validation experiments.

Criteria	Cutoff	Random Control (%)	Linkage Interval (%)	Genome-wide Scan (%)
Precision	90%	28.28 (0.42)	33.45 (1.57)	5.34 (0.33)
	80%	27.42 (0.76)	32.06 (1.44)	4.79 (0.47)
	70%	27.43 (1.06)	29.59 (0.93)	3.97 (0.74)
	60%	25.68 (1.04)	30.11 (1.10)	3.58 (0.58)
	50%	25.85 (0.73)	28.47 (0.89)	5.35 (1.22)

Mean Rank Ratio	90%	10.90 (0.78)	8.98 (0.64)	10.16 (0.81)
	80%	11.23 (0.84)	9.37 (0.92)	10.38 (1.02)
	70%	11.68 (1.21)	10.09 (1.05)	10.61 (0.69)
	60%	12.37 (0.97)	9.21 (1.01)	10.76 (0.80)
	50%	13.47 (0.55)	9.72 (0.98)	10.95 (0.78)

AUC	90%	89.92 (0.30)	90.83 (0.58)	89.87 (0.61)
	80%	89.60 (0.22)	90.26 (0.43)	89.66 (0.29)
	70%	89.15 (0.17)	89.40 (0.64)	89.46 (0.48)
	60%	88.48 (0.59)	90.01 (0.51)	89.32 (0.34)
	50%	87.31 (0.28)	89.89 (0.46)	89.16 (0.75)

The small domain-domain interaction network composed of the PDB part of the DOMINE database and the diffusion kernel are used to obtain the results. Results for random controls are mean (standard deviation) of 10 validation runs.

In order to study how known domain-disease associations for other diseases contribute to the inference of domains that are associated with the query disease, we keep 10%, 20%, 30%, 40%, and 50% disease phenotypes that have the highest similarity scores to the query disease, respectively, and we repeat the leave-one-out validation processes, using the diffusion kernel measure and the small domain-domain interaction network that is composed of the PDB part of the DOMINE database. Results (Table 5) show that our method is robust in this experiment, in the sense that the values of the three evaluation criteria do not change significantly. For example, in the cross-validation for random controls, the changes of precisions are no more than 6.64%, the changes of mean rank ratios are no more than 1.15%, and the changes of AUC scores are no more than 1.20%. However, we also notice that the performance of our approach tends to drop when more phenotypes with lower similarity scores are included. For example, in the experiment, our approach achieves the highest performance when keeping only 10% phenotypes which have the highest similarity scores to the query phenotype and the lowest performance when keeping 50% of the most similar phenotypes, although the drop in performance is small. We also repeat the above analysis using the large domain-domain interaction network that includes the entire DOMINE database and the high-confidence part of the InterDom database, and we obtain similar results [Additional file 2: Supplemental Table S1]. From these results, we conclude that seed domain-disease associations in which the diseases have high phenotype similarity scores with the query disease have main contributions in the prioritization procedure.

**Table 5:** Contributions of seed domain-disease associations based on leave-one-out cross-validation experiments.

Criteria	Cutoff	Random Control (%)	Linkage Interval (%)	Genome-wide Scan (%)
Precision	10%	37.82 (0.72)	42.29	8.16
	20%	34.55 (1.03)	38.39	8.63
	30%	32.85 (0.77)	37.52	7.79
	40%	32.78 (0.97)	36.98	7.79
	50%	31.18 (0.62)	34.50	6.16

Mean Rank Ratio	10%	8.83 (0.06)	7.87	7.96
	20%	8.80 (0.07)	7.92	7.95
	30%	9.23 (0.03)	8.47	8.33
	40%	9.64 (0.07)	8.61	8.74
	50%	9.98 (0.06)	8.86	9.09

AUC	10%	92.14 (0.12)	94.08	91.75
	20%	92.16 (0.10)	93.91	91.83
	30%	91.71 (0.10)	93.46	91.42
	40%	91.33 (0.08)	92.63	91.08
	50%	90.94 (0.06)	92.30	90.66

The small domain-domain interaction network composed of the PDB part of the DOMINE database and the diffusion kernel are used to obtain the results. Results for random controls are mean (standard deviation) of 10 validation runs.

### *Ab initio *inference of domain-disease and gene-disease associations

Above we have used several large scale leave-one-out cross-validation experiments to evaluate the performance and robustness of the proposed domainRBF approach. However, it might be argued that a disease may be associated with more than one domain and that, consequently, the inclusion of domains already known to be associated with the query disease in the calculation of the domain proximity profile may ease the identification of novel associations. Following this line of reasoning, we demonstrate the capability of the proposed domainRBF approach in the prediction of novel associations for query diseasesby performing the following *ab initio *inference experiments. For each query disease, we calculate domain proximity profiles with the exclusion of all domains that are known to be associated with the disease (i.e., as if genetic bases of the disease were completely unknown), apply the domainRBF method to score candidate domains, and then prioritize the candidates. We again perform random control, linkage interval, and genome-wide validation experiments and evaluate the performance of our approach in terms of precision, mean rank ratio, and AUC scores. We perform this *ab initio *inference using the large network that is composed of entire interactions in DOMINE and high-confidence interactions in InterDom (with diffusion kernel), and we summarize the results in Table 6.

**Table 6:** *Ab initio *inference of domain-disease associations.

	Random Control (%)	Linkage Interval (%)	Genome-wide Scan (%)
Precision	22.11 (0.45)	24.39	3.90
Mean Rank Ratio	17.34 (0.07)	17.07	16.45
AUC	83.43 (0.07)	84.62	83.27

In comparison with the results in Table 2, we find that the performance of the domainRBF approach slightly drops (less than 4%). For example, for the random control validation, the precision is almost the same, the mean rank ratio drops from 14.96% to 17.34%, and the AUC score from 85.82% to 83.43%. For the linkage interval validation, the precision drops from 27.56% to 24.39%, the mean rank ratio from 14.12% to 17.07%, and the AUC score from 86.68% to 84.62%. For the genome-wide validation, the precision even increases slightly from 3.47% to 3.90%, while the mean rank ratio drops from 14.11% to 16.45% and the AUC score from 85.72% to 83.27%. In other words, for a query disease of interest, our approach is capable of inferring novel associations between domains and the disease without prior knowledge about genetic bases of the disease. This characteristic of our approach is of great importance, because the genetic bases for about half of the diseases in the OMIM database are still unknown [47].

We also study the contribution of seed domain-disease associations by keeping a fraction of disease phenotypes that have the highest similarity scores to the query disease and repeating *ab initio *prediction experiments. We observe from the results [Additional file 2: Supplemental Tables 2 and 3] that seed domain-disease associations in which the diseases have high phenotype similarity scores with the query disease have main contributions in the prioritization procedure. We further study whether the *ab initio *prediction tends to give higher ranks to domains that occur more frequently in human proteins. We merge the frequency of occurrence of domains in all human proteins into 11 bins (0-10, 11-20, 21-30, 31-40. 41-50, 51-60, 61-70, 71-80, 81-90. 91-100, 101 and above), and we look at how ranks of domains that are known to be associated with diseases distribute in different bins. From the results [Additional file 1: Supplemental Figure S2], we see that the median of mean ranks of such domains do not show much change for different bins, indicating that our method is not biased towards common domains.

**Figure 7:**
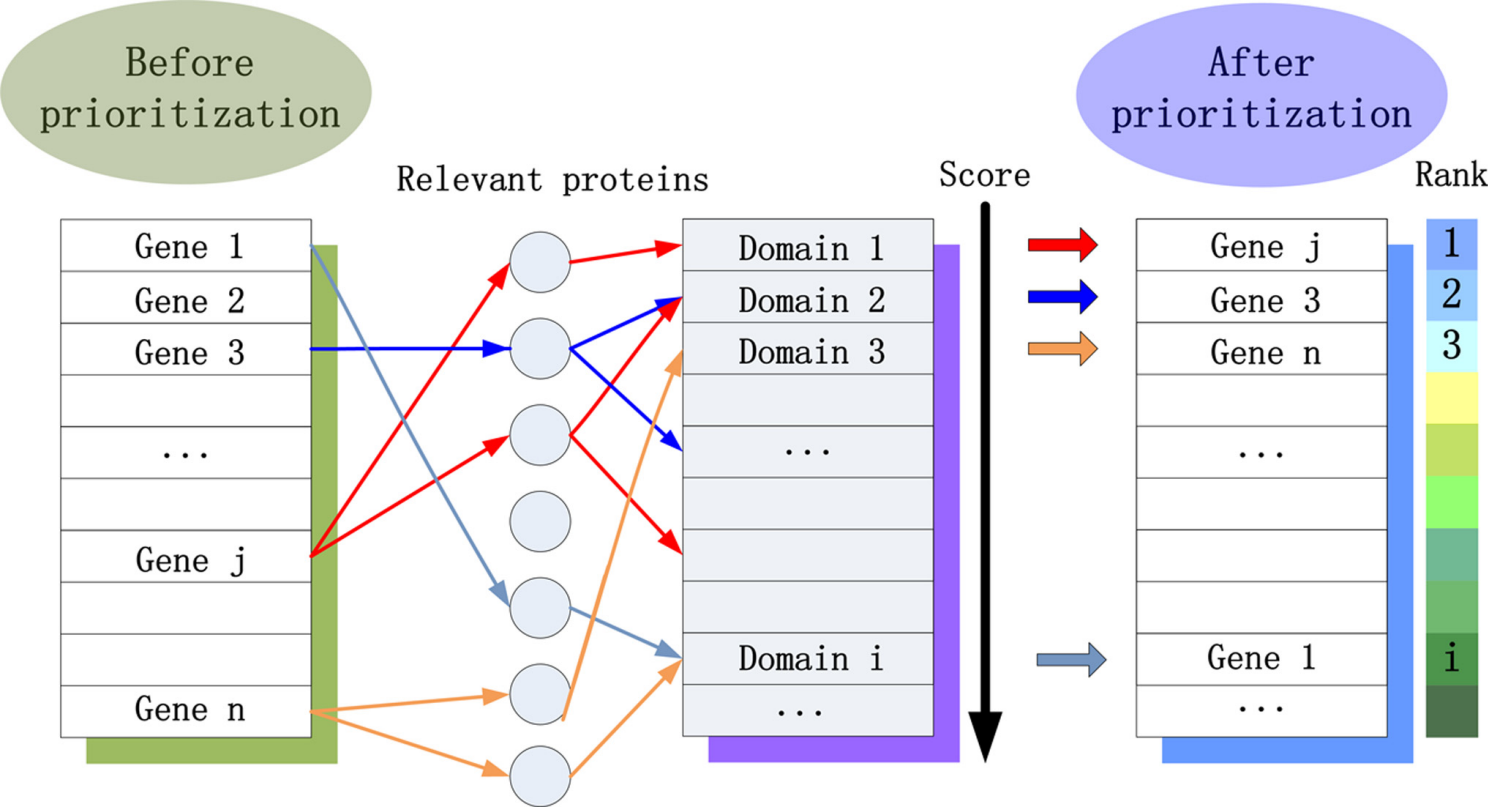
**Scheme of inferring gene-disease associations from domain-disease associations**.

Inspired by the success of *ab initio *inference of domains and diseases, we further propose the following application of the proposed domainRBF approach in the inference of genes that are associated with diseases, by combining predicted domain-disease associations and known domain-protein relations. As shown in Figure 7, given a query disease and a gene whose products (proteins) are usually composed of several protein domains, we look at corresponding Bayes factors of these domains and define an association score that measures the strength of association between the gene and the disease as the maximum among these Bayes factors. Then, given a set of candidate genes, we are able to obtain association scores for the genes and further rank the genes according to their scores.

We validate our gene prioritization approach using known gene-disease associations extracted from the BioMart database [48,49]. After ruling out diseases that do not exist in our phenotype similarity network and genes whose products do not have domain annotation, we obtain 2,847 associations between 1,737 genes and 1,875 diseases. We then prioritize each of these genes that are known to be associated with some diseases (disease genes) against a total of 14,944 genes whose products have domain annotations. Results show that in 207 out of the 2,847 associations, the known disease genes rank first in the candidate list of 14,944 genes, obtaining a precision of 7.27%, as well as a fold enrichment of 1,087 (It should be noted that fold enrichment is defined in existing literature [32] as follows: for a method that is able to rank known disease genes among the top *a*% of all candidates in *b*% validation runs, the fold enrichment is *b/a *on average). In other words, our domainRBF approach can also be effectively used as an intermediate step to infer associations between genes and diseases.

### Genome-wide evidence of associations between domains and common human diseases

The identification of susceptible single nucleotide polymorphisms (SNPs) conferring risk for common human diseases is one of the main tasks of genome-wide association studies (GWAS). Since the study that identified the association of complement factor H (CFH) with age-related macular degeneration (AMD) in 2005, over 450 GWAS have been performed and more than 2,000 susceptible SNPs or genetic loci have been reported [50]. With these resources, it is of interest to to determine the extent to which the genome-wide *ab initio *inference of associations between domains and diseases are consistent with these GWAS results.

Given a disease of interest, we collect from SNPedia [51,52] or other relevant literatures a list of reported susceptible SNPs, and we see how many of these SNPs appear within 5 Mbp of the domains that are ranked in the top 10 in our genome-wide *ab initio *inference, which uses all domains in the domain-domain interaction network as candidates. We further implement the following permutation test to check whether the number of such susceptible SNPs is significantly enriched within these regions, as

1. Count the number of reported SNPs that appear within 5 Mbp of domains that are ranked among the top 10 in the genome-wide *ab initio *inference. Record this number as *N*_0_.

2. For the *i*-th permutation, select 10 domains at random from all domains in the genome-wide *ab initio *inference. Count the number of reported SNPs that appear within 5 Mbp of these domains. Record this number as *N*_*i*_.

3. Repeat the above random selection *M *times (*M *= 10,000 in our study). Count the number of times that *N*_*i *_(*i *= 1,...,*N*) is greater than or equal to *N*_0_. Record this number as *m*.

4. Calculate a *p*-value as *p *= *m*/*M*.

The null hypothesis in the above permutation test is that the number of the reported susceptible SNPs within 5 Mbp regions of the high ranking domains (top 10) is not different from that of randomly selected domains. Therefore, a small *p*-value indicates that the reported susceptible SNPs tend to be closer to the high ranking domains. In other words, high ranking domains are more likely to be associated with the disease under investigation.

We then select four disease examples (Type 1 diabetes, Type 2 diabetes, Crohn's disease, and Breast cancer), apply the above permutation test method to these diseases, and analyze the results in detail. We choose these four diseases because they are common and have GWAS results available. It has been shown that diabetes had affected 2.8% of the population worldwide by 2000 [53], with type 2 diabetes as the most common form of this disease [54]. It is also known that Crohn's disease affects 0.2% to 0.1% people within the UK [55], and that breast cancer is the most common type of non-skin cancer in women and the fifth most common cause of cancer death [56,57].

#### Type 1 Diabetes

Type 1 diabetes, formerly called juvenile diabetes or insulin-dependent diabetes, is a condition in which pancreatic *β *cell destruction usually leads to absolute insulin deficiency [58]. The genetic susceptibility of Type 1 diabetes is strongly associated with HLA-DQ and DR on chromosome 6, but genetic factors on other chromosomes such as the insulin gene on chromosome 11 and the cytotoxic T-lymphocyte antigen gene on chromosome 2 may modulate disease risk [59]. In our study, we compile from SNPedia 48 reported susceptible SNPs, and 25 of them are found to be within 5Mbp regions of 6 domains (i.e., NACHT, Recep_L_domain, Collagen, HNF-1A_C, CARD, and FGF) that are ranked among the top 10. We present the detailed list of these domains and SNPs [Additional file 2: Supplemental Table S4]. In summary, we observe 3 times that a susceptible SNP locates inside a domain (once in each of Recep_L_domain, Collagen, and HNF-1A_C, respectively), 15 times that a susceptible SNP locates within 1 Mbp upstream or downstream of a domain, and 44 times that a susceptible SNP locates within 5 Mbp region of a domain. The permutation test, as described above, yields a *p*-value of 0.0313, which is smaller than 0.05. From these results we conjecture that domains ranked among the top 10 do, indeed, tend to be closer to, or even include, known susceptible SNPs for this disease.

In addition, we also examine the 4 domains that are not close to susceptible SNPs reported by GWAS. For domain HNF-1B_C (PF04812), we notice that Urhammer *et al*. [60] have pointed out that mutations and polymorphisms in HNF-1 cause the type 3 form of maturity-onset diabetes of the young (MODY3), and for domain HNF-1_N (PF04814), mutations and the common polymorphism Ala/Val in position 98 of HNF-1 also cause MODY3. It is known that MODY3 is a kind of monogenic diabetes, which is different from type 1 diabetes that involves more complex combinations of causes involving multiple genes and environmental factors (i.e., polygenic). However, most commonly MODY3 acts like a very mild version of type 1 diabetes, with continued partial insulin production and normal insulin sensitivity [61]. Therefore, domain HNF-1B_C (PF04812) and domain HNF-1_N (PF04814), which are highly ranked in terms of our approach, may also be closer to, even include, known susceptible SNPs for this disease.

#### Type 2 Diabetes

Type 2 diabetes, formerly called adult-onset diabetes or noninsulin-dependent diabetes, is the most common form of diabetes. It usually begins with insulin resistance, a condition in which fat, muscle, and liver cells do not use insulin properly [62]. Numerous SNPs have been associated with (slightly) increased risk for type-2 diabetes [63,64], but they only marginally improve the odds of predicting whether an individual will get type-2 diabetes based on the traditional clinical characteristics combining age, sex and weight [65]. In our study, we compile from SNPedia and reference [64] a total of 53 reported susceptible SNPs, and 24 of them are found to be within 5 Mbp regions of 7 domains (i.e., HNF-1B_C, IF_tail, Sulfatase, Collagen, Alk_phosphatase, Pkinase_Tyr, and FGF) that are ranked among top 10. We present the detailed list of these domains and SNPs [Additional file 2: Supplemental Table S5]. In summary, we observe 47 times that a susceptible SNP locates within 1 Mbp upstream or downstream of a domain, and 102 times that a susceptible SNP locates within 5 Mbp region of a domain. The permutation test yields a *p*-value of 0.0363, which is smaller than 0.05. From these results we conjecture that domains ranked among the top 10 do, indeed, tend to be closer to known susceptible SNPs for this disease.

In addition, we also examine the 3 domains that are not close to susceptible SNPs reported by GWAS. We notice that both domains HNF-1A_C (PF04813) and HNF-1_N (PF04814) contain mutations that may cause the type 3 form of maturity-onset diabetes of the young (MODY3), as pointed out by Urhammer *et al*. [60]. Although type 2 diabetes may share some characteristics in common with MODY3, no direct evidence has yet been found to demonstrate that these two domains cause type 2 diabetes.

#### Crohn's Disease

Crohn's disease, a chronic inflammatory disorder of the gastrointestinal tract, is thought to result from the combination of effect of environmental factors and genetic predisposition [66,67]. Recently, genome-wide association studies have made notable progress in the study of this disease, with the number of confirmed associated loci increasing from two to more than ten [68,69]. In our study, we compile from SNPedia 36 reported susceptible SNPs, and 29 of them are found to be within 5 Mbp regions of 8 domains (i.e., Sulfatase, NACHT, Collagen, Crystall, Pkinase_Tyr, CARD, Gla, and Hormone_1) that are ranked among top 10. We present the detailed list of these domains and SNPs [Additional file 2: Supplemental Table S6]. In summary, we observe 5 times that a susceptible SNP locates inside a domain (3 times in NACHT and 2 times in Pkinase_Tyr), 21 times that a susceptible SNP locates within 1 Mbp upstream or downstream of a domain, and 49 times that a susceptible SNP locates within 5 Mbp region of a domain. The permutation test yields a *p*-value of 0.0029, which is far smaller than 0.05. From these results we conjecture that domains ranked among the top 10 do, indeed, tend to be closer to, or even include, known susceptible SNPs for this disease.

#### Breast Cancer

Breast cancer, the most common malignancy in women in the Western world [70], exhibits a characteristic of familial clustering [70,71]. Although little is known currently to explain the familial clustering of breast cancer, a large amount of susceptible genes and SNPs of this disease have been recently reported, including the well-known high breast cancer risk in BRCA1 and BRCA2 mutation carriers as well as the risk for breast cancer in certain rare syndromes caused by mutations in TP53, STK11, PTEN, CDH1, NF1 or NBN [70]. In our study, we compile from SNPedia 60 reported susceptible SNPs, and 38 of them are found to be within 5 Mbp regions of 7 domains (i.e., Sulfatase, Pkinase_Tyr, Crystall, FGF, Collagen, NACHT, and Recep_L_domain) that are ranked among the top 10. We present the detailed list of these domains and SNPs [Additional file 2: Supplemental Table S7]. In summary, we observe 6 times that a susceptible SNP locates inside a domain (all in Pkinase_Tyr), 21 times that a susceptible SNP locates within 1 Mbp upstream or downstream of a domain, and 80 times that a susceptible SNP locates within 5 Mbp region of a domain. The permutation test yields a *p*-value of 0.0159, which is smaller than 0.05. From these results we conjecture that domains ranked among the top 10 do, indeed, tend to be closer to, or even include, known susceptible SNPs for this disease.

#### Contributions of seed domain-disease associations in the analysis of the four diseases

For each of the four diseases examples, we further evaluate the contribution of seed domain-disease associations by keeping 10%, 20%, 30%, 40%, and 50% disease phenotypes that have the highest similarity scores to the query disease, obtaining a rank list of all domains, and then using the permutation test to check whether the number of known susceptible SNPs is still significantly enriched around the top ranking domains. As shown in [Additional file 2: Supplemental Table S8], we find that all resulting *p*-values are smaller than 0.05, and are also numerically close to those obtained using all phenotypes. We therefore conjecture that our approach is robust to the seed domain-disease associations in the inference for these disease examples.

### A predicted landscape of domain-disease associations

With the above validation results demonstrating the possibility of recovering the associations between protein domains and disease phenotypes, we further apply the domainRBF approach to all available protein domains and human disease phenotypes and predict a genome-wide landscape of the associations between protein domains and human disease phenotypes. There are a total of 5,080 phenotypes in the phenotype similarity network and 5,490 protein domains in the domain-domain interaction network (the union of the entire DOMINE and InterDom network). For each phenotype, we perform a prioritization of all domains with the use of the domainRBF approach (using the diffusion kernel measure). The prioritization results, together with a freely accessible web interface, are provided at http://bioinfo.au.tsinghua.edu.cn/domainRBF/domain. All domains on the webpage are linked to the DOMINE database and the InterDom database, from which further information can be obtained.

**Figure 8:**
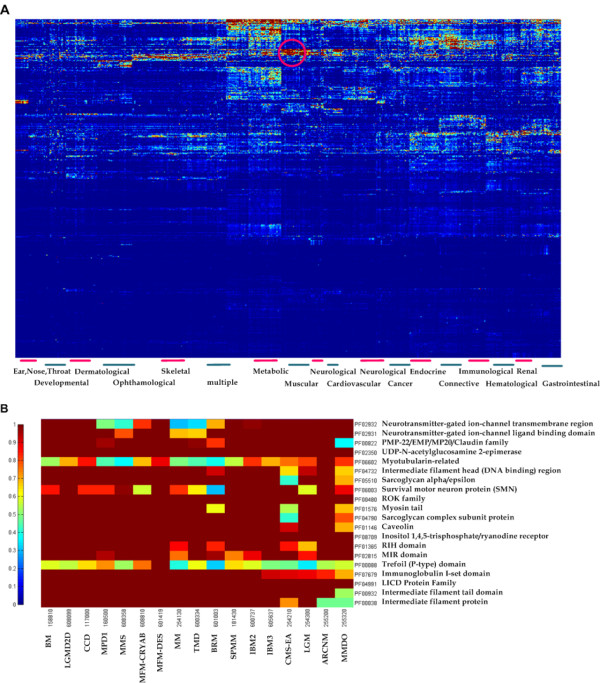
**Modular organization of the predicted landscape of human disease phenotypes**. (A) Two-way hierarchical clustering heat map for the landscape of domain-phenotype associations. (B) Zoomed-in plot of the pink circle region in the heat map, involving 17 muscular diseases and 20 related protein domains.

On the basis of the above prioritization results, we aggregate the Bayes factors between all the 5,490 domains and 1,145 phenotypes, and obtain a matrix of altogether 6,286,050 elements. Here we first make a log (base 10) transform of original matrix, and then implement clustering while removing the rows in which the values are all smaller than 0.1. Since phenotypes clustered together generally have similar molecular basis, or share significant genetic overlaps [32], we implement a two-way hierarchical clustering [72], to identify interesting areas where large values of Bayes factors are highly enriched. The clustering result is demonstrated in the form of a heat map, as shown in Figure 8(A). We then manually inspect and annotate each of the phenotype clusters with one of the 22 disorder classes based on the physiological system affected [73]. Through clustering, many highly scored blocks or regions are formed in the heat map, each of which represents a set of functionally related domains implicated in a set of genetically overlapping phenotypes [32]. Specifically, we take the region in the pink circle as an example, which is enlarged in Figure 8(B). Phenotypes in the region selected are enriched with diseases related to the muscle system, and domains are also conjectured to share similar functions with adjacent domains in the same region.

We further apply the above prioritization method to all human disease phenotypes and obtain a landscape of gene-phenotype associations that include 5,080 disease phenotypes and 14,944 human genes. The prioritization results, together with a freely accessible web interface, are provided at http://bioinfo.au.tsinghua.edu.cn/domainRBF/gene/. All genes on the webpage are linked to the Ensembl database [74], from which further information can be obtained.

## Discussion and Conclusions

In this paper, we studied the problem of identifying domains that are associated with human inherited diseases under a prioritization framework. We proposed an approach called domainRBF from the perspective of Bayesian regression, verified its superior performance through three large-scale cross-validation experiments, and demonstrated the robustness of this approach via a series of permutation tests. We further proposed to perform *ab initio *inference of domain-disease associations and gene-disease associations. Finally, we calculated a landscape between 5,490 protein domains and 5,080 disease phenotypes.

In comparison with previous studies that rely on phenotype similarity and protein-protein interaction data to infer gene-disease associations [32], our approach can achieve higher resolution in pinpointing susceptibility functional units in the genome, essentially because a domain is only a fraction of a protein and is typically small in size (ranging between 40 and 700 residues [75] with an average of approximately 100 residues [76]). Moreover, as demonstrated in the Results section, our approach can also be used as an intermediate step in the inference of gene-disease associations.

However, our method has the following limitations. First, our method can only be applied to diseases that are included in phenotype similarity data and domains that are included in domain interaction data. In the case of phenotype similarity, a possible solution would involve the development of a visualization and annotation system such as the one in [77] that can associate a new disease to a standard vocabulary and then calculate similarities for the new disease. In the case of domain interaction data, a possible solution would involve the development of effective computational methods to predict domain-domain interactions.

Second, our method currently only considers conjugate priors in the Bayesian regression model. Although such formulation results in analytic solutions and thus alleviates the computational burden in the calculation of Bayes factors, it is known that the specification of prior is intrinsically complicated and subjective [44]. The main consideration is that the posterior mean and variance should not depend on the units in which the disease similarities are measured and should also be invariant to the shift of the response variable. To meet these requirements, the use of the Jeffreys prior [78] could be considered, and a Markov chain Monte Carlo (MCMC) approach could be adopted for the calculation of the marginal likelihood.

Our approach can be further studied from the following aspects. First, in addition to the domain-domain interaction network, information such as annotations of Pfam domains in the Gene Ontology (GO) can also provide a means for calculating similarities between domains. Recently, methods for calculating semantic similarities between GO terms have been packed into user-friendly software [79]. It is therefore possible to calculate pair-wise semantic similarities between every two domains and then use this similarity profile with our domainRBF model to infer associations between domains and human inherited diseases.

Second, it is conceptually straightforward to extend the domainRBF model to infer interactive effects of multiple domains on a query disease. For example, given a query disease and a set of candidate domains, we can enumerate all two-way combinations of the domains and then use the DomainRBF model to infer possible associations between the disease and interactions of two domains. Nevertheless, such brute force method is computationally intensive and not quite feasible in application to the study of three-way or even higher order interactive effects of candidate domains.

Third, with the accumulation of publicly available data in genome-wide association (GWA) studies, we can consider the integration of our method and GWA studies. For example, given a disease of interest and a set of candidate domains, we can prioritize the candidate domains using our method and obtain the ranks of the domains. On the other hand, given *p*-values of SNPs in a GWA study, we can obtain the statistical significance of candidate domains in the GWA study by combining the *p*-values of SNPs located in the domains and then prioritize the candidates to obtain their ranks. With these two ranks, we can resort to statistical methods, such as the one described in [8], to obtain a single rank for each candidate domain.

## Competing interests

The authors declare that they have no competing interests.

## Authors' contributions

WZ derived the model, implemented the method, collected the results and drafted the manuscript. YC participated in the design of the study. FS designed the research and revised the manuscript. RJ designed the research, drafted, and revised the manuscript. All authors read and approved the final manuscript.

## Supplementary Material

Additional file 1 **Supplemental Figures**. Supplemental Figure S1 shows the results of a series of permutation test using the large domain-domain interaction network. Supplemental Figure S2 shows the mean ranks for domains with different frequency of occurrence in human proteins. Click here for file

Additional file 2 **Supplemental Tables**. Supplemental Table S1 lists contributions of seed domain-disease associations (leave-one-out cross-validation experiments using the large domain-domain interaction network). Supplemental Table S2 lists contributions of seed domain-disease associations (*ab initio *prediction experiments using the small domain-domain interaction network). Supplemental Table S3 lists contributions of seed domain-disease associations (*ab initio *prediction experiments using the large domain-domain interaction network). Supplemental Table S4 lists the genome-wide evidence of associations between domains and type 1 diabetes. Supplemental Table S5 lists the genome-wide evidence of associations between domains and type 2 diabetes. Supplemental Table S6 lists the genome-wide evidence of associations between domains and Crohn's disease. Supplemental Table S7 lists the genome-wide evidence of associations between domains and breast cancer. Supplemental Table S8 lists contributions of seed domain-disease associations in the analysis of the four disease examples. Click here for file
