# Supplementary material for: DomainRBF: a Bayesian regression approach to the prioritization of candidate domains for complex diseases
Source: BMC Syst Biol. 2011 Apr 19;5:55. doi: 10.1186/1752-0509-5-55 (PMC3108930; doi:10.1186/1752-0509-5-55)
Supplement: Additional file 1 — Supplemental Figures. Supplemental Figure S1 shows the results of a series of permutation test using the large domain-domain interaction network. Supplemental Figure S2 shows the mean ranks for domains with different frequency of occurrence in human proteins. [file 1752-0509-5-55-S1.PDF]

Additional File 1

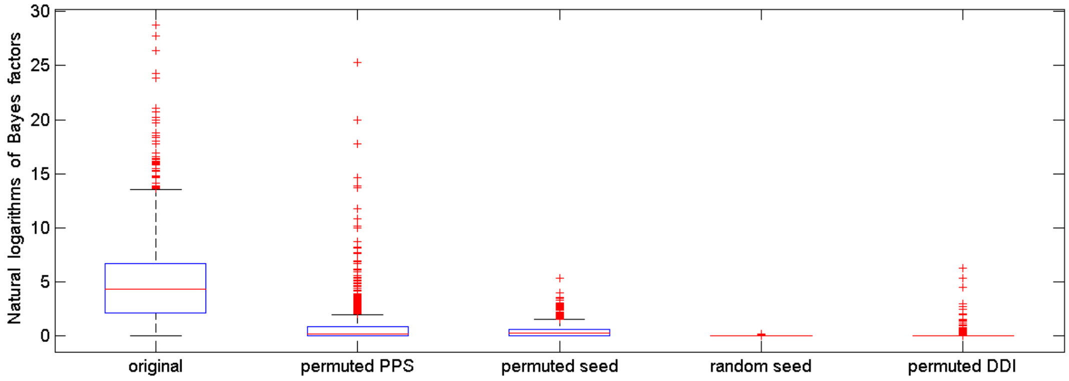

Supplemental Figure 1: Bayes factors of the original and permuted data. "original", "permuted PPS", "permuted seed", "random seed", and "permuted DDI" denote the results obtained using the original data, permuted phenotype similarity profile, permuted domain-disease associations, randomly selected seed domains, and permuted domain-domain interaction network, respectively. The large domain-domain interaction network composed of the entire DOMINE database and high-confidence interactions in the InterDom database, together with the diffusion kernel, is used to obtain the results.

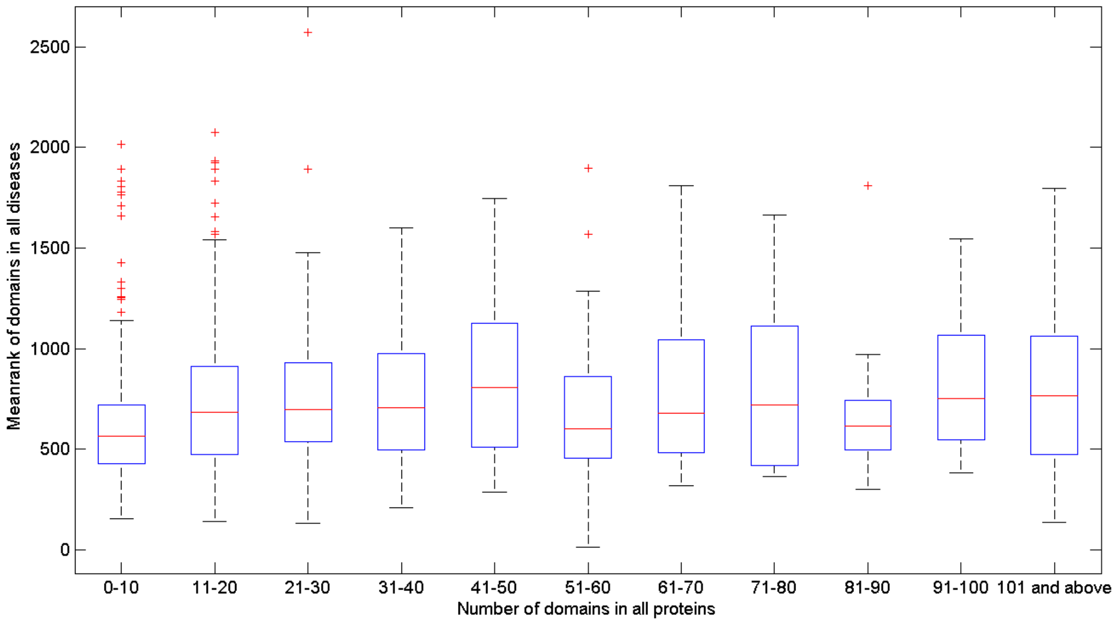

Supplemental Figure 2: Mean ranks of domains in all diseases. Results show the relationship between mean ranks of domains and frequencies of occurrence of domains in all human proteins, based on *ab initio* prediction experiments.
