# Supplementary material for: DomainRBF: a Bayesian regression approach to the prioritization of candidate domains for complex diseases
Source: BMC Syst Biol. 2011 Apr 19;5:55. doi: 10.1186/1752-0509-5-55 (PMC3108930; doi:10.1186/1752-0509-5-55)
Supplement: Additional file 2 — Supplemental Tables. Supplemental Table S1 lists contributions of seed domain-disease associations (leave-one-out cross-validation experiments using the large domain-domain interaction network). Supplemental Table S2 lists contributions of seed domain-disease associations (ab initio prediction experiments using the small domain-domain interaction network). Supplemental Table S3 lists contributions of seed domain-disease associations (ab initio prediction experiments using the large domain-domain interaction network). Supplemental Table S4 lists the genome-wide evidence of associations between domains and type 1 diabetes. Supplemental Table S5 lists the genome-wide evidence of associations between domains and type 2 diabetes. Supplemental Table S6 lists the genome-wide evidence of associations between domains and Crohn's disease. Supplemental Table S7 lists the genome-wide evidence of associations between domains and breast cancer. Supplemental Table S8 lists contributions of seed domain-disease associations in the analysis of the four disease examples. [file 1752-0509-5-55-S2.PDF]

Additional File 2

Supplemental Table 1
Contributions of seed domain-disease associations based on leave-one-out cross-validation experiments.

Criteria	Cutoff	Random Control (%)	Linkage Interval (%)	Genome-wide Scan (%)
Precision	10%	32.35 (0.67)	33.77	5.33
	20%	27.76 (0.95)	30.04	5.08
	30%	25.43 (0.43)	28.17	5.02
	40%	24.54 (0.49)	28.67	4.77
	50%	23.64 (0.42)	26.44	4.71
Mean Rank Ratio	10%	11.72 (0.07)	9.82	10.85
	20%	12.80 (0.05)	11.05	11.95
	30%	13.66 (0.04)	11.23	12.82
	40%	13.94 (0.07)	11.50	13.08
	50%	14.47 (0.07)	12.11	13.60
AUC	10%	89.13 (0.07)	91.86	88.93
	20%	88.03 (0.05)	90.01	87.85
	30%	87.16 (0.05)	89.14	86.99
	40%	86.88 (0.07)	88.06	86.73
	50%	86.34 (0.07)	87.28	86.23

The large domain-domain interaction network composed of the entire DOMINE database and high-confidence interactions in the InterDom database, together with the diffusion kernel, is used to obtain the leave-one-out cross-validation results. Results for random controls are mean (standard deviation) of 10 validation runs.

Supplemental Table 2
Contributions of seed domain-disease associations based on *ab initio* prediction experiments.

Criteria	Cutoff	Random Control (%)	Linkage Interval (%)	Genome-wide Scan (%)
Precision	10%	39.53 (0.51)	44.71	15.10
	20%	36.97 (0.90)	40.20	13.04
	30%	35.46 (0.65)	39.94	13.70
	40%	34.85 (0.74)	38.93	12.95
	50%	33.14 (0.51)	36.04	12.85
Mean Rank Ratio	10%	13.59 (0.06)	11.51	12.76
	20%	13.21 (0.05)	11.60	12.40
	30%	13.21 (0.05)	12.48	12.37
	40%	13.58 (0.06)	12.67	12.73
	50%	13.72 (0.08)	12.98	12.85
AUC	10%	87.24 (0.16)	90.97	86.58
	20%	87.56 (0.10)	89.95	86.79
	30%	87.51 (0.13)	89.79	86.70
	40%	87.21 (0.12)	89.04	86.43
	50%	87.05 (0.09)	88.13	86.41

The small domain-domain interaction network composed of the PDB part of the DOMINE database, together with the diffusion kernel, is used to obtain the results. Results for random controls are mean (standard deviation) of 10 validation runs.

Supplemental Table 3
Contributions of seed domain-disease associations based on *ab initio* prediction experiments.

Criteria	Cutoff	Random Control (%)	Linkage Interval (%)	Genome-wide Scan (%)
Precision	10%	31.32 (0.79)	34.15	7.81
	20%	27.66 (0.52)	30.99	7.56
	30%	25.66 (0.68)	28.92	7.31
	40%	25.16 (0.68)	28.41	7.00
	50%	24.05 (0.49)	26.35	6.34
Mean Rank Ratio	10%	15.31 (0.05)	13.08	14.49
	20%	16.06 (0.08)	13.11	15.20
	30%	16.38 (0.06)	14.38	15.52
	40%	16.44 (0.04)	14.49	15.60
	50%	17.09 (0.09)	15.75	16.25
AUC	10%	85.49 (0.05)	87.70	85.29
	20%	84.74 (0.08)	86.68	84.60
	30%	84.41 (0.06)	85.41	84.29
	40%	84.34 (0.05)	85.30	84.20
	50%	83.68 (0.09)	84.73	83.56

The large domain-domain interaction network composed of the entire DOMINE database and high-confidence interactions in the InterDom database, together with the diffusion kernel, is used to obtain the results. Results for random controls are mean (standard deviation) of 10 validation runs.

Supplemental Table 4
Genome-wide evidence of associations between domains and type 1 diabetes.

Rank	Domain	Chr	Region(Mb)	SNP	Position(Mb)	Distance
1	PF04812	--	--	--	--	>5Mb
2	PF00884	--	--	--	--	>5Mb
3	PF05729	11	7.0417-7.09276	rs689	2.18222	4.85948Mb up
			0.27857-0.285304	rs689	2.18222	1.89692Mb down
		16	10.971-11.0236	rs12708716	11.1799	0.156249Mb down
4	PF01030	12	56.4739-56.4971	rs11171739	56.4706	0.003267Mb up
				rs2292239	56.4822	inside
5	PF00245	--	--	--	--	>5Mb
6	PF04814	--	--	--	--	>5Mb
7	PF01391	5	33.9873-34.1246	rs6897932	35.8746	1.74994Mb down
		3	48.6015-48.6327	rs1799864	46.3992	2.2023Mb up
				rs333	46.4149	2.18656Mb up
				rs6441961	46.3524	2.24912Mb up
		22	37.5762-37.5954	rs229541	37.5913	inside
		2	189.839-189.877	rs7574865	191.965	2.08716Mb down
		3	186.56-186.576	rs1464510	188.113	1.5363Mb down
			161.215-161.222	rs17810546	159.665	1.54955Mb up
				rs9811792	159.697	1.5176Mb up
		6	33.1305-33.1603	rs2040410	32.5309	0.599582Mb up
				rs7454108	32.6815	0.448975Mb up
				rs9272346	32.6044	0.526086Mb up
				rs2296336	33.6367	0.476384Mb down
		2	189.897-190.045	rs7574865	191.965	1.92003Mb down
8	PF04813	22	37.8864-37.9155	rs229541	37.5913	0.295082Mb up
		2	163.124-163.175	rs1990760	163.124	inside
		5	40.8413-40.8602	rs6897932	35.8746	4.96671Mb up
9	PF00619	3	191.86-192.486	rs1464510	188.113	3.74713Mb up
		4	123.748-123.819	rs17388568	123.329	0.418501Mb up
				rs2069763	123.377	0.370381Mb up
				rs6822844	123.509	0.238442Mb up
10	PF00167	12	10.7715-10.8269	rs11052552	9.85596	0.91558Mb up
			14.7656-14.8495	rs11052552	9.85596	4.90961Mb up
		3	185.001-185.207	rs1464510	188.113	2.90567Mb down
		15	75.0744-75.0955	rs3825932	79.2354	4.13991Mb down
		2	98.33-98.3563	rs917997	103.071	4.71424Mb down
		6	93.9497-94.1293	rs11755527	90.9582	2.99151Mb up
			91.2233-91.2968	rs11755527	90.9582	0.265061Mb up
		12	53.8743-53.8933	rs11171739	56.4706	2.57735Mb down
				rs2292239	56.4822	2.58891Mb down
		3	49.9244-49.9413	rs1799864	46.3992	3.52523Mb up
				rs333	46.4149	3.50949Mb up
				rs6441961	46.3524	3.57205Mb up

		11	6.62496-6.6321	rs689	2.18222	4.44274Mb up
		3	184.28-184.3	rs1464510	188.113	3.81236Mb down
		6	30.8488-30.8679	rs2040410	32.5309	1.66294Mb down
				rs7454108	32.6815	1.81355Mb down
				rs9272346	32.6044	1.73644Mb down
				rs2296336	33.6367	2.76873Mb down

"Rank" denotes the rank of the corresponding domain in the *ab initio* inference of domain-disease associations. "Domain" denotes the Pfam ID of the domain. "Chr" denotes the chromosome at which the domain locates. "Region" denotes predicted regions that may include susceptible SNPs (Mb means 10E6 base pairs). "SNP" denotes reported susceptible SNP from the literature or databases. "Position" denotes position of the susceptible SNP. "Distance" denotes the distance from the susceptible SNP to the predicted domain region.

Supplemental Table 5
Genome-wide evidence of associations between domains and type 2 diabetes.

Rank	Domain	Chr	Region(Mb)	rsSNP	Position(Mb)	Distance
1	PF04812	17	36.0464-36.1052	rs17705177	33.1976	2.84879Mb up
2	PF04813	--	--	--	--	>5Mb
3	PF04814	--	--	--	--	>5Mb
4	PF00932	12	65.1072-65.1532	rs7961581	69.9494	4.79614Mb down
5	PF00884	12	48.3667-48.3983	rs12304921	51.3575	2.95926Mb down
				rs1153188	53.3853	4.98698Mb down
		10	16.5557-16.564	rs12779790	12.368	4.18773Mb up
		3	186.56-186.576	rs4402960	186.994	0.418137Mb down
		8	120.079-120.119	rs13266634	118.254	1.82548Mb up
				rs10282940	118.257	1.82244Mb up
			121.072-121.384	rs13266634	118.254	2.81806Mb up
				rs10282940	118.257	2.81501Mb up
		3	15.4916-15.5633	rs1801282	12.3681	3.12352Mb up
				rs17036101	12.2528	3.23879Mb up
				rs13071168	12.2504	3.24119Mb up
6	PF01391	3	186.256-186.264	rs4402960	186.994	0.729898Mb down
7	PF00030	--	--	--	--	>5Mb
8	PF00245	12	56.4739-56.4971	rs1153188	53.3853	3.08863Mb up
		4	1.79503-1.8106	rs4580722	6.42231	4.61172Mb down
		3	185.001-185.207	rs4402960	186.994	1.7875Mb down
		15	75.0744-75.0955	rs2930291	72.3919	2.68254Mb up
		6	43.044-43.1295	rs9472138	43.9197	0.790283Mb down
		9	27.1091-27.2302	rs10811661	22.1241	4.98505Mb up
				rs7020996	22.1196	4.98957Mb up
		3	12.6251-12.7057	rs1801282	12.3681	0.256975Mb up
				rs17036101	12.2528	0.372255Mb up
				rs13071168	12.2504	0.374653Mb up
		12	53.8743-53.8933	rs12304921	51.3575	2.51674Mb up
				rs1153188	53.3853	0.489017Mb up
		17	37.8444-37.8849	rs17705177	33.1976	4.64675Mb up
		1	65.2989-65.4322	rs4655595	66.5588	1.12657Mb down
		10	43.5725-43.6258	rs9326506	43.3886	0.183912Mb up
		3	184.28-184.3	rs4402960	186.994	2.69419Mb down
		1	64.2397-64.6472	rs4655595	66.5588	1.91158Mb down
9	PF07714	3	191.86-192.486	rs4402960	186.994	4.86529Mb up
		4	123.748-123.819	rs7659604	122.885	0.8629Mb up
10	PF00167	10	17.2703-17.2796	rs12779790	12.368	4.90224Mb up
		12	53.3427-53.3467	rs12304921	51.3575	1.98511Mb up
				rs1153188	53.3853	0.038579Mb down
		17	16.7441-16.7492	rs11868035	17.7151	0.965904Mb down

		rs2236513	17.7474	0.998169Mb down
		rs6502618	17.5005	0.751287Mb down
		rs1889018	17.7347	0.985543Mb down
		rs2297508	17.7153	0.96612Mb down
12	49.6875-49.6925	rs12304921	51.3575	1.66508Mb down
		rs1153188	53.3853	3.6928Mb down
	52.7538-52.7613	rs12304921	51.3575	1.39625Mb up
		rs1153188	53.3853	0.623954Mb down
	52.6443-52.6523	rs12304921	51.3575	1.28671Mb up
		rs1153188	53.3853	0.732926Mb down
	52.627-52.6427	rs12304921	51.3575	1.26941Mb up
		rs1153188	53.3853	0.742554Mb down
	52.9377-52.9469	rs12304921	51.3575	1.58015Mb up
		rs1153188	53.3853	0.438332Mb down
	52.7716-52.7794	rs12304921	51.3575	1.41405Mb up
		rs1153188	53.3853	0.605846Mb down
	52.7877-52.8002	rs12304921	51.3575	1.43019Mb up
		rs1153188	53.3853	0.585087Mb down
	52.5628-52.5858	rs12304921	51.3575	1.20524Mb up
		rs1153188	53.3853	0.799479Mb down
	53.0685-53.0742	rs12304921	51.3575	1.71098Mb up
		rs1153188	53.3853	0.311072Mb down
	53.291-53.2989	rs12304921	51.3575	1.93343Mb up
		rs1153188	53.3853	0.086395Mb down
	53.2327-53.2428	rs12304921	51.3575	1.8752Mb up
		rs1153188	53.3853	0.142485Mb down
	52.6956-52.7029	rs12304921	51.3575	1.33811Mb up
		rs1153188	53.3853	0.682316Mb down
	52.818-52.8283	rs12304921	51.3575	1.46044Mb up
		rs1153188	53.3853	0.556954Mb down
	52.8623-52.8676	rs12304921	51.3575	1.50476Mb up
		rs1153188	53.3853	0.517694Mb down
	53.2003-53.2083	rs12304921	51.3575	1.84279Mb up
		rs1153188	53.3853	0.176928Mb down
	52.9596-52.9676	rs12304921	51.3575	1.60206Mb up
		rs1153188	53.3853	0.417654Mb down
	52.9794-52.9953	rs12304921	51.3575	1.62183Mb up
		rs1153188	53.3853	0.389971Mb down
	52.7081-52.7152	rs12304921	51.3575	1.35054Mb up
		rs1153188	53.3853	0.670081Mb down
	53.0383-53.046	rs12304921	51.3575	1.6808Mb up
		rs1153188	53.3853	0.339304Mb down
	53.1619-53.1711	rs12304921	51.3575	1.8044Mb up

	rs1153188	53.3853	0.214158Mb down
52.8404-52.8459	rs12304921	51.3575	1.48289Mb up
	rs1153188	53.3853	0.539353Mb down
53.2152-53.2281	rs12304921	51.3575	1.85769Mb up
	rs1153188	53.3853	0.157184Mb down
53.0014-53.0123	rs12304921	51.3575	1.64381Mb up
	rs1153188	53.3853	0.37292Mb down
52.9084-52.9142	rs12304921	51.3575	1.55082Mb up
	rs1153188	53.3853	0.47102Mb down
53.1835-53.1899	rs12304921	51.3575	1.82593Mb up
	rs1153188	53.3853	0.195371Mb down
18.3301-18.3353	rs11868035	17.7151	0.615019Mb up
	rs2236513	17.7474	0.582754Mb up
	rs6502618	17.5005	0.829636Mb up
	rs1889018	17.7347	0.59538Mb up
	rs2297508	17.7153	0.614803Mb up
53.0834-53.0972	rs12304921	51.3575	1.72587Mb up
	rs1153188	53.3853	0.288016Mb down
52.881-52.8872	rs12304921	51.3575	1.52342Mb up
	rs1153188	53.3853	0.498082Mb down
52.6797-52.6853	rs12304921	51.3575	1.32216Mb up
	rs1153188	53.3853	0.699964Mb down

"Rank" denotes the rank of the corresponding domain in the ab initio inference of domain-disease associations. "Domain" denotes the Pfam ID of the domain. "Chr" denotes the chromosome at which the domain locates. "Region" denotes predicted regions that may include susceptible SNPs (Mb means 10E6 base pairs). "SNP" denotes reported susceptible SNP from the literature or databases. "Position" denotes position of the susceptible SNP. "Distance" denotes the distance from the susceptible SNP to the predicted domain region.

Supplemental Table 6
Genome-wide evidence of associations between domains and Crohn's disease.

Rank	Domain	Chr	Region(Mb)	rsSNP	Position(Mb)	Distance
1	PF00884	5	149.676-149.683	rs1000113	150.24	0.557551Mb down
				rs13361189	150.223	0.540862Mb down
				rs4958847	150.24	0.557062Mb down
2	PF05729	16	50.7311-50.767	rs17221417	50.7396	inside
				rs2066844	50.7459	inside
				rs2076756	50.7569	inside
3	PF01391	10	105.791-105.846	rs10883365	101.288	4.50328Mb up
		3	48.6015-48.6327	rs9858542	49.702	1.06928Mb down
				rs1128535	49.8664	1.23369Mb down
				rs2230590	49.9361	1.3034Mb down
				rs1062633	49.9249	1.29224Mb down
		2	238.233-238.323	rs10210302	234.159	4.07381Mb up
				rs2241880	234.183	4.04928Mb up
		6	33.1305-33.1603	rs9469220	32.6583	0.472148Mb up
4	PF00030	7	151.126-151.138	rs7807268	148.258	2.86787Mb up
5	PF04812	--	--	--	--	>5Mb
6	PF07714	5	149.493-149.535	rs1000113	150.24	0.704653Mb down
				rs13361189	150.223	0.687964Mb down
				rs4958847	150.24	0.704164Mb down
		19	40.6977-40.7215	rs1800471	41.8589	1.1374Mb down
		8	11.3515-11.4221	rs11362	6.7354	4.61611Mb up
				rs1800972	6.73542	4.61609Mb up
		19	48.9885-49.0164	rs8111071	46.3074	2.68112Mb up
		1	233.464-233.521	rs5051	230.85	2.61364Mb up
			65.2989-65.4322	rs11805303	67.6755	2.24333Mb down
				rs11209026	67.706	2.27377Mb down
				rs1004819	67.6702	2.23803Mb down
				rs2201841	67.6942	2.26202Mb down
				rs10889677	67.7251	2.29293Mb down
		3	49.9244-49.9413	rs9858542	49.702	0.222452Mb up
				rs1128535	49.8664	0.058043Mb up
				rs2230590	49.9361	inside
				rs1062633	49.9249	inside
		19	41.7251-41.7677	rs8111071	46.3074	4.53974Mb down
				rs1800471	41.8589	0.091206Mb down
		5	149.433-149.493	rs1000113	150.24	0.747141Mb down
				rs13361189	150.223	0.730452Mb down
				rs4958847	150.24	0.746652Mb down
		1	64.2397-64.6472	rs11805303	67.6755	3.02833Mb down
				rs11209026	67.706	3.05878Mb down
				rs1004819	67.6702	3.02303Mb down
				rs2201841	67.6942	3.04702Mb down

		1	64.2397-64.6472	rs10889677	67.7251	3.07794Mb down
		6	30.8488-30.8679	rs9469220	32.6583	1.79038Mb down
7	PF00619	19	48.7114-48.7531	rs8111071	46.3074	2.40395Mb up
		5	40.8413-40.8602	rs17234657	40.4015	0.439777Mb up
				rs1992660	40.4151	0.426219Mb up
				rs1992662	40.3939	0.447434Mb up
8	PF00594	19	50.0846-50.0943	rs8111071	46.3074	3.77718Mb up
9	PF00103	6	22.2875-22.2977	rs6908425	20.7287	1.55875Mb up
10	PF02758	--	--	--	--	>5Mb

"Rank" denotes the rank of the corresponding domain in the *ab initio* inference of domain-disease associations. "Domain" denotes the Pfam ID of the domain. "Chr" denotes the chromosome at which the domain locates. "Region" denotes predicted regions that may include susceptible SNPs (Mb means 10E6 base pairs). "SNP" denotes reported susceptible SNP from the literature or databases. "Position" denotes position of the susceptible SNP. "Distance" denotes the distance from the susceptible SNP to the predicted domain region.

Supplemental Table 7
Genome-wide evidence of associations between domains and Breast cancer.

Rank	Domain	Chr	Region(Mb)	rsSNP	Position(Mb)	Distance
1	PF00884	20	46.2851-46.4154	rs3918242	44.636	1.64912Mb up
2	PF07714	19	3.77797-3.80181	rs713041	1.10661	2.67135Mb up
				rs757229	1.10211	2.67585Mb up
		5	180.029-180.077	rs351855	176.52	3.50826Mb up
		12	10.7715-10.8269	rs34330	12.8707	2.0438Mb down
		10	123.238-123.358	rs2981578	123.34	inside
				rs3135718	123.354	inside
				rs7895676	123.334	inside
				rs2981582	123.352	inside
				rs1219648	123.346	inside
		12	14.7656-14.8495	rs34330	12.8707	1.89488Mb up
		13	28.8745-29.0692	rs766173	32.9065	3.83725Mb down
				rs4987117	32.9142	3.845Mb down
				rs1799954	32.9146	3.84536Mb down
				rs11571746	32.9451	3.87588Mb down
				rs11571747	32.9452	3.87594Mb down
				rs4987047	32.9535	3.8843Mb down
				rs11571833	32.9726	3.90339Mb down
				rs1801426	32.9729	3.90365Mb down
				rs144848	32.9067	3.8375Mb down
		2	222.283-222.439	rs13387042	217.906	4.37691Mb up
		13	28.5774-28.6747	rs766173	32.9065	4.23175Mb down
				rs4987117	32.9142	4.23951Mb down
				rs1799954	32.9146	4.23986Mb down
				rs11571746	32.9451	4.27038Mb down
				rs11571747	32.9452	4.27044Mb down
				rs4987047	32.9535	4.2788Mb down
				rs11571833	32.9726	4.2979Mb down
				rs1801426	32.9729	4.29816Mb down
				rs144848	32.9067	4.232Mb down
		17	7.90599-7.92366	rs1042522	7.57947	0.326516Mb up
			37.8444-37.8849	rs1799950	41.2465	3.36157Mb down
				rs4986850	41.2455	3.36056Mb down
				rs2227945	41.2441	3.35921Mb down
				rs16942	41.244	3.35909Mb down
				rs1799966	41.2231	3.33818Mb down
		5	176.514-176.525	rs351855	176.52	inside
		11	6.62496-6.6321	rs3817198	1.90901	4.71596Mb up
		17	7.28437-7.29309	rs1042522	7.57947	0.28638Mb down
		2	212.24-213.404	rs13387042	217.906	4.50227Mb down

		22	31.6082-31.6761	rs17879961	29.1211	2.48714Mb up
		6	30.8488-30.8679	rs361525	31.5431	0.675168Mb down
3	PF00030	22	25.5958-25.6033	rs17879961	29.1211	3.51776Mb down
			26.9952-27.0141	rs17879961	29.1211	2.10703Mb down
		7	151.126-151.138	rs3218536	152.346	1.20811Mb down
		2	219.855-219.858	rs13387042	217.906	1.94908Mb up
		22	27.0179-27.0266	rs17879961	29.1211	2.09445Mb down
			25.6155-25.6278	rs17879961	29.1211	3.49325Mb down
4	PF03542	--	--	--	--	>5Mb
5	PF00167	5	44.3036-44.3888	rs4415084	44.6625	0.273731Mb down
		19	0.639895-0.643703	rs713041	1.10661	0.462912Mb down
				rs757229	1.10211	0.458411Mb down
		10	103.53-103.536	rs12762549	101.621	1.90931Mb up
		17	7.34269-7.34826	rs1042522	7.57947	0.231216Mb down
6	PF01391	5	177.665-178.018	rs351855	176.52	1.14438Mb up
		10	105.791-105.846	rs12762549	101.621	4.17027Mb up
		17	43.0371-43.0456	rs1799950	41.2465	1.79058Mb up
				rs4986850	41.2455	1.79159Mb up
				rs2227945	41.2441	1.79293Mb up
				rs16942	41.244	1.79306Mb up
				rs1799966	41.2231	1.81397Mb up
		4	15.3416-15.4478	rs2056116	13.01	2.33157Mb up
		22	29.6018-29.6556	rs17879961	29.1211	0.480753Mb up
		6	33.1305-33.1603	rs1801270	36.652	3.4917Mb down
				rs3176336	36.6488	3.48854Mb down
				rs361525	31.5431	1.58736Mb up
7	PF01847	--	--	--	--	>5Mb
8	PF05729	17	5.40472-5.48783	rs1042522	7.57947	2.09164Mb down
		16	57.0234-57.1174	rs3803662	52.5863	4.43707Mb up
		16	50.7311-50.767	rs3803662	52.5863	1.81935Mb down
		11	0.27857-0.285304	rs3817198	1.90901	1.6237Mb down
9	PF01030	17	41.8435-41.8564	rs1799950	41.2465	0.597009Mb up
				rs4986850	41.2455	0.598019Mb up
				rs2227945	41.2441	0.59936Mb up
				rs16942	41.244	0.59949Mb up
				rs1799966	41.2231	0.620396Mb up
		12	12.6288-12.7153	rs34330	12.8707	0.155378Mb down
		5	172.195-172.198	rs351855	176.52	4.32205Mb down
		10	112.258-112.271	rs12255372	114.809	2.5376Mb down
		12	89.7418-89.7463	rs2268578	91.5012	1.7549Mb down
		22	31.048-31.0639	rs17879961	29.1211	1.92695Mb up
		11	1.57528-1.59346	rs3817198	1.90901	0.315547Mb down
10	PF00782	--	--	--	--	>5Mb

“Rank” denotes the rank of the corresponding domain in the *ab initio* inference of domain-disease associations. “Domain” denotes the Pfam ID of the domain. “Chr” denotes the chromosome at which the domain locates. “Region” denotes predicted regions that may include susceptible SNPs (Mb means 10E6 base pairs). “SNP” denotes reported susceptible SNP from the literature or databases. “Position” denotes position of the susceptible SNP. “Distance” denotes the distance from the susceptible SNP to the predicted domain region.

Supplemental Table 8
***p*-values of the permutation tests for the four disease examples.**

	Type 1 Diabetes	Type 2 Diabetes	Crohn's Diseases	Breast Cancer
10%	0.0391	0.0445	0.0032	0.0194
20%	0.0331	0.0458	0.0029	0.0163
30%	0.0378	0.0402	0.0029	0.0139
40%	0.0371	0.0472	0.0028	0.0152
50%	0.0337	0.0455	0.0015	0.0141

Results are obtained using 10%, 20%, 30%, 40%, and 50% phenotypes that have the highest similarity scores to the query disease.
